# ALKBH5/YTHDF2 Axis Regulates Osteogenic Differentiation Through Mediating the m^6^A Modification of ELK1

**DOI:** 10.1155/ije/2669506

**Published:** 2026-01-30

**Authors:** Huan Yu, Ting Ruan, Yongxing Peng

**Affiliations:** ^1^ Trauma Emergency Department, Jiangxi Provincial People’s Hospital, The First Affiliated Hospital of Nanchang Medical College, No. 152 Aiguo Road, Nanchang, 330006, China, jxsrmyy.cn; ^2^ Obstetrical Department, Jiangxi Maternal and Child Health Hospital, Maternal and Child Health Hospital of Nanchang Medical College, No. 318 Bayi Avenue, Nanchang, 330000, China, jxsfybjyy.cn

**Keywords:** ALKBH5, ELK1, m^6^A modification, osteogenic difference, osteoporosis, YTHDF2

## Abstract

This study aimed to investigate the role of N^6^‐methyladenosine (m^6^A) methylation in osteogenic differentiation during osteoporosis (OP). Serum specimens were obtained from 25 individuals diagnosed with OP and 25 age‐matched healthy controls. In parallel, MC3T3‐E1 preosteoblastic cells were employed for in vitro functional assays. Expression levels of m^6^A‐associated genes were quantified using qPCR. Osteogenic potential was evaluated by measuring ALP activity with an ALP assay kit and by assessing matrix mineralization through Alizarin Red S staining. RIP and dual‐luciferase reporter assays were performed to elucidate the molecular interactions involved. To corroborate the in vitro observations, an ovariectomized (OVX) mouse model of OP was established for in vivo validation. The results revealed a significant downregulation of AlkB Homolog 5 (ALKBH5) in both serum samples from OP patients and MC3T3‐E1 cells undergoing osteogenic differentiation. Moreover, enforced expression of ALKBH5 suppressed osteogenic differentiation in these cells. Mechanistically, ELK1 was found to be a key downstream effector of ALKBH5. Additionally, YTH domain family protein 2 (YTHDF2) was demonstrated to function as the m^6^A reader that specifically recognizes the ALKBH5‐mediated demethylation site on ELK1 mRNA. Rescue experiments confirmed that ELK1 overexpression or YTHDF2 knockdown promoted osteogenic differentiation, whereas these effects were abolished by ALKBH5 overexpression or ELK1 silencing. In OVX mice, ALKBH5 knockdown mitigated bone loss, improved bone strength, and restored ELK1 expression. Notably, ELK1 inhibition reversed the protective effects of YTHDF2 knockdown on bone loss and mechanical strength in OVX mice. In conclusion, ALKBH5/YTHDF2 axis might be involved in osteogenic differentiation via regulating ELK1 (a key downstream effector), which might provide a new insight for OP treatment.

## 1. Introduction

Osteoporosis (OP) is a systemic skeletal disease marked by reduced bone mass and a disrupted microarchitecture, increasing fracture risk [1, 2]. It poses a major public health burden, especially in aging populations [3]. OP arises from an imbalance between osteoblast‐driven bone formation and osteoclast‐mediated resorption, governed by intricate molecular mechanisms, including transcriptional and post‐transcriptional regulation [4, 5].

Emerging research has established that m^6^A RNA methylation is a critical regulator of skeletal homeostasis. Dysregulation of central m^6^A effectors, including the methyltransferase METTL3 and the eraser protein FTO, is observed to directly influence two pivotal processes: the osteoblastic differentiation of progenitor cells and the activity of bone‐resorbing osteoclasts, as indicated in recent studies [6, 7]. Among these regulators, AlkB Homolog 5 (ALKBH5), a well‐characterized m^6^A “eraser,” has garnered growing interest; however, its exact function in the context of OP is still not fully elucidated. Recent investigations have started to shed light on ALKBH5’s role in skeletal homeostasis. A study by Huang et al. [8] demonstrates that the demethylase ALKBH5 inhibits osteoblast differentiation under osteoporotic conditions. This effect is achieved through its m^6^A eraser activity on VDAC3 mRNA, which subsequently intensifies cellular senescence triggered by etoposide. Similarly, Li et al. [9] indicate that ALKBH5 suppresses mesenchymal stem cell commitment to osteogenesis through its functional interaction with protein arginine methyltransferase 6 (PRMT6). Despite these advances, the downstream targets and broader regulatory networks of ALKBH5 in OP pathogenesis remain largely unexplored, warranting further mechanistic investigations to uncover its therapeutic potential.

ELK1, an ETS family transcription factor belonging to the ternary complex factor (TCF) subfamily [10], has been increasingly recognized as a key modulator of bone metabolism. Accumulating data indicate that ELK1 critically influences osteoblast differentiation and the dynamic process of bone remodeling. Wang et al. [11] report that downregulating microRNA‐139‐3p mitigates its inhibitory impact on osteoblast differentiation and reduces apoptosis in MC3T3‐E1 cells exposed to simulated microgravity. This regulatory effect involves targeting the transcription factor ELK1. Furthermore, ELK1 has been implicated in osteogenic differentiation in other bone‐related disorders, such as chronic periodontitis [12]. Notably, prior work has demonstrated that ELK1 promotes prostate cancer progression by upregulating YTH domain family protein 1 (YTHDF1), which enhances the m^6^A‐dependent translation of Polo‐like kinase 1 [13]. Despite this insight, the potential involvement of ALKBH5 in osteogenic differentiation through the modulation of ELK1 has not yet been investigated. Elucidating the regulatory network of ELK1—especially its interplay with m^6^A methylation and noncoding RNAs—is crucial for advancing precision therapeutics targeting osteoporosis (OP) and related metabolic bone disorders.

The ovariectomized (OVX) mouse model is extensively employed in OP research because it faithfully mimics the bone loss observed in postmenopausal women [14, 15]. Here, we aimed to define the role of ALKBH5 in OP pathogenesis by integrating in vivo analyses in OVX mice with in vitro osteogenic differentiation assays using MC3T3‐E1 cells, focusing specifically on ALKBH5‐mediated m^6^A RNA methylation. Our findings support a model in which ALKBH5 acts as a potent suppressor of osteogenic differentiation by erasing m^6^A marks on ELK1 mRNA, thereby downregulating the expression and function of this pivotal downstream effector.

## 2. Materials and Methods

### 2.1. Patients’ and Samples’ Collection

Twenty‐five OP patients treated in our hospital were selected as the study subjects, along with 25 healthy individuals undergoing physical examinations during the same period. All participants provided written informed consent after being fully informed about the study. The research protocol was reviewed and approved by the Medical Ethics Committee of Jiangxi Provincial People’s Hospital, which is also the First Affiliated Hospital of Nanchang Medical College (Approval No. KT042). Clinical data were collected from all enrolled individuals. Morning venous blood samples (∼5 mL) were obtained from all participants after an overnight fast. Serum was then isolated from these samples for further analysis.

### 2.2. Dot Blot Assay

Serum or cell lysates were diluted in PBS, heat‐denatured (65°C, 10 min), and 2 μL spotted onto a nitrocellulose membrane, which was air‐dried. After blocking with 5% nonfat milk in TBST (1 h, RT), the membrane was incubated overnight at 4°C with anti‐m^6^A antibody (1:5000; Abcam) and then with HRP‐conjugated secondary antibody (1:2000; Abcam) for 1 h. Signals were detected using ECL, quantified by ImageJ, and normalized to methylene blue staining.

### 2.3. Cell Culture and Induction

The MC3T3‐E1 pre‐osteoblastic cell line (Chinese Academy of Sciences, Shanghai, China) was cultured in DMEM with 5% FBS and 1% penicillin/streptomycin at 37°C, 5% CO_2_ and induced for 14 days with osteogenic induction medium (OIM) (50 μg/mL ascorbic acid, 10 mM β‐glycerophosphate,) upon reaching 80% confluence.

### 2.4. Cell Transfection

siRNAs (si‐ALKBH5, si‐YTHDF2, si‐ELK1) and overexpression plasmids for ALKBH5, YTH family members, and ELK1, along with respective controls, were synthesized by GenePharma Technology Co., Ltd. (Shanghai, China). MC3T3‐E1 cells (2 × 10^5^/well in six‐well plates) were transfected at 70%–80% confluence using Lipofectamine 3000 (Thermo Fisher Scientific, USA).

### 2.5. Cell Viability Evaluation

MC3T3‐E1 cells seeded in 96‐well plates were incubated with 10 μL of CCK‐8 (Beyotime Biotech, Shanghai, China) reagent at 37°C for 4 h, and absorbance was measured at 450 nm using a microplate reader.

### 2.6. Alkaline Phosphatase (ALP) Activity and Alizarin Red S (ARS) Staining Detection

ALP activity was assessed using a fluorescence kit (Jiancheng Institute, Nanjing, China) per the manufacturer’s instructions and normalized to cell count and protein content. Mineralized nodules were visualized by ARS staining: differentiated MC3T3‐E1 cells were fixed (4% formaldehyde, 15 min), stained with 2% Alizarin Red (30 min, RT), rinsed, and imaged at 450 nm.

### 2.7. Quantitative Real‐Time PCR (qPCR)

Total RNA was extracted using the RNAsimple kit (TIANGEN), and 1 μg was reverse‐transcribed with BeyoRT III Kit (Beyotime). qPCR was performed using BeyoFast SYBR Green Mix (Beyotime), with expression normalized to GAPDH via the 2^−ΔΔCt^ method. Primers (GenePharma) are listed in Table 1.

**Table 1:** Primer sequences used in qPCR.

Name	Forward (5′‐3′)	Reverse (5′‐3′)
ALKBH5 (h)	CGG​CGA​AGG​CTA​CAC​TTA​CG	CCA​CCA​GCT​TTT​GGA​TCA​CCA
METTL3 (h)	TTG​TCT​CCA​ACC​TTC​CGT​AGT	CCA​GAT​CAG​AGA​GGT​GGT​GTA​G
METTL14 (h)	GAA​CAC​AGA​GCT​TAA​ATC​CCC​A	TGT​CAG​CTA​AAC​CTA​CAT​CCC​TG
FTO (h)	ACT​TGG​CTC​CCT​TAT​CTG​ACC	TGT​GCA​GTG​TGA​GAA​AGG​CTT
WTAP (h)	CTT​CCC​AAG​AAG​GTT​CGA​TTG​A	TCA​GAC​TCT​CTT​AGG​CCA​GTT​AC
RBM15 (h)	ACG​ACC​CGC​AAC​AAT​GAA​G	GGA​AGT​CGA​GTC​CTC​ACC​AC
ALKBH5 (m)	GCG​CGG​TCA​TCA​ACG​ACT​A	ATC​AGC​AGC​ATA​CCC​ACT​GAG
ELK1 (m)	TTG​TGT​CCT​ACC​CAG​AGG​TTG	GCT​ATG​GCC​GAG​GTT​ACA​GA
YTHDC1 (m)	GAG​AAT​GGA​GTC​TAC​TGA​CAC​CA	ACA​GAC​GAA​TTT​TTC​GAT​CAG​CA
YTHDC2 (m)	GAA​GAT​CGC​CGT​CAA​CAT​CG	GCT​CTT​TCC​GTA​CTG​GTC​AAA
YTHDF1 (m)	ACA​GTT​ACC​CCT​CGA​TGA​GTG	GGT​AGT​GAG​ATA​CGG​GAT​GGG​A
YTHDF2 (m)	GAG​CAG​AGA​CCA​AAA​GGT​CAA​G	CTG​TGG​GCT​CAA​GTA​AGG​TTC
YTHDF3 (m)	GAT​CAG​CCT​ATG​CCA​TAT​CTG​AC	CCC​CTG​GTT​GAC​TAA​AAA​CAC​C
GAPDH (h)	TGT​GGG​CAT​CAA​TGG​ATT​TGG	ACA​CCA​TGT​ATT​CCG​GGT​CAA​T
GAPDH (m)	AAT​GGA​TTT​GGA​CGC​ATT​GGT	TTT​GCA​CTG​GTA​CGT​GTT​GAT

### 2.8. Western Blot

Total protein was extracted using RIPA buffer with protease inhibitors, quantified by BCA assay (Thermo Fisher), and 30 μg loaded onto 10% SDS–PAGE gels, followed by transfer to PVDF membranes. Membranes were blocked with 5% nonfat milk in TBST for 1 h at room temperature and incubated overnight at 4°C with primary antibodies against ALKBH5, YTHDC1/2, YTHDF1/2/3, ELK1 (all 1:1000; Abcam), or β‐actin (1:2000; Abcam). After TBST washes, a secondary antibody (1:5000; Beyotime) was applied for 1 h. Signals were detected using the ECL substrate (Thermo Fisher), quantified by ImageJ.

### 2.9. Methylated RNA Immunoprecipitation (MeRIP)‐qPCR

Total RNA from MC3T3‐E1 cells was fragmented and incubated with anti‐m^6^A antibody, normal IgG (negative control), and affinity beads using the EpiQuik CUT&RUN kit (EpigenTek, Farmingdale, NY, USA). Immunoprecipitated and input RNAs were reverse‐transcribed, and qPCR was performed with gene‐specific primers.

### 2.10. RIP Assay

Cells were lysed in RIP buffer and incubated with anti‐YTHDF2 or IgG magnetic beads (4°C, 4 h). After elution and Proteinase K digestion (55°C, 30 min), RNA was purified and analyzed by qPCR for ELK1.

### 2.11. Bioinformatics Analysis

ALKBH5‐interacting proteins were identified using the GeneMANIA database, followed by pathway enrichment analysis via FunRich. ELK1 m^6^A sites were predicted using the SRAMP online tool.

### 2.12. Luciferase Assay

A dual‐luciferase assay (Promega, Madison, USA) was used to test the ALKBH5/YTHDF2‐mediated m^6^A regulation of ELK1. Wild‐type and mutant ELK1 3′UTRs were cloned into pGL3 vectors and cotransfected into MC3T3‐E1 cells with ALKBH5/YTHDF2 (or empty) plasmids and pRL‐TK for normalization. Luciferase activity was measured 48 h post‐transfection, with appropriate positive and negative controls included.

### 2.13. RNA Stability

MC3T3‐E1 cells were treated with Actinomycin D (0.5 μg/mL); RNA was collected at 0, 1, 2, 4, and 8 h, and ELK1 mRNA levels were assessed by qPCR.

### 2.14. Animal Study

All procedures were approved by Jiangxi Provincial People’s Hospital (KT055). Female BALB/c mice (9 weeks, ∼20 g) were housed under standard conditions. OVX was performed under isoflurane anesthesia via bilateral lumbar incisions and ovary excision [16]; sham mice underwent identical surgery without ovariectomy. One week postsurgery, mice in the sh‐NC, sh‐ALKBH5, sh‐YTHDF2, or sh‐ELK1 groups received tail vein injections of 100 μL adenovirus (1 × 10^9^ TU/mL; Hanbio Biotechnology Co., Ltd., Shanghai, China) expressing the respective shRNA. Eight weeks later, femurs were harvested for micro‐CT analysis (μCT‐80, voxel size: 9 μm; 55 kV, 70 mA). The region of interest (ROI) was defined 0.3–0.6 mm distal to the growth plate [16]. Bone parameters—including BMD, BV/TV, Tb.N, Tb.Th, Tb.Sp, and TBPf—were quantified from 3D reconstructions.

### 2.15. Histological Staining

Mouse femurs were fixed, EDTA‐decalcified, and paraffin‐embedded; 5‐μm sections underwent H & E or TRAP staining—H & E: hematoxylin (5 min), HCl–ethanol differentiation, eosin (30 s); TRAP: incubation at 37°C for 1 h, followed by hematoxylin counterstaining. Slides were analyzed by light microscopy for morphology and TRAP^+^ osteoclasts.

### 2.16. Biomechanical Test

Femur mechanical properties were assessed via three‐point bending on an electronic universal testing machine (Testing Machine Institute, Changchun, China) [17]. Samples were supported 7 mm apart, and a vertical load was applied at the midpoint at 1 mm/min until fracture. The load–displacement curve was used to calculate stiffness (K, N/mm) and elastic modulus (MPa).

### 2.17. Statistical Analysis

Statistical analyses were performed using GraphPad Prism 8.0 (GraphPad Software, Inc., La Jolla, CA, USA). Two‐group comparisons used the unpaired Student’s *t* test; multiple groups were analyzed by one‐way ANOVA with Tukey’s HSD *post hoc* test. Data from ≥ 3 independent experiments are shown as mean ± SD; *p* < 0.05 (two‐tailed) was considered significant.

## 3. Results

### 3.1. Clinical Information

As shown in Table 2, OP patients and healthy controls did not differ significantly in sex distribution or BMI. However, OP patients were older: 98% were aged ≥ 50 years compared to 72% in the control group, while only 4% were < 50 years vs. 28% in controls. Additionally, the OP patient group exhibited a higher proportion of individuals with a history of bone fracture (76% vs. 0%), medication use (100% vs. 84%), and complications (100% vs. 76%).

**TABLE 2:** Clinical information.

Subjects	*n*	Normal (*n* = 25)	Patients (*n* = 25)	*X* ^2^	*p* value
Age (years)				5.357	0.021^∗^
< 50	8	7 (28%)	1 (4%)		
≥ 50	42	18 (72%)	24 (96%)		
Sex				3.125	0.077
Male	18	12 (48%)	6 (24%)		
Female	32	13 (52%)	19 (76%)		
BMI (kg/m^2^)				0.085	0.771
< 24	31	15 (60%)	16 (64%)		
≥ 24	19	10 (40%)	9 (36%)		
The history of bone fracture				30.650	< 0.001^∗∗^
YES	19	0 (0%)	19 (76%)		
NO	31	25 (100%)	6 (24%)		
Medicine use				4.348	0.037^∗^
YES	46	21 (84%)	25 (100%)		
NO	4	4 (16%)	0 (0%)		
Complications				6.818	0.009^∗∗^
YES	44	19 (76%)	25 (100%)		
NO	6	6 (24%)	0 (0%)		

^∗^ *p* < 0.05.

^∗∗^ *p* < 0.01.

### 3.2. ALKBH5 Expression Was Downregulated in OP Patients’ Serum and in Osteogenic Differentiated MC3T3‐E1 Cells

A significant reduction in global m^6^A levels was observed in OP patient sera relative to controls (Figure 1(a)). During the osteogenic induction of MC3T3‐E1 cells, m^6^A abundance increased markedly compared to undifferentiated cells (Figure 1(b)). Given this dysregulation, we evaluated the expression of major m^6^A regulators by qPCR. Notably, only ALKBH5 was significantly upregulated in serum from OP patients (Figures 1(c), 1(d), 1(e), 1(f), 1(g), 1(h)), yet downregulated during osteogenic differentiation of MC3T3‐E1 cells (Figure 1(i)). These results implicated ALKBH5 as a potential modulator of osteoblast differentiation.

Figure 1ALKBH5 expression was downregulated in the serum of OP patients and in osteogenic differentiated MC3T3‐E1 cells. (a) Total m^6^A level in the serum samples of OP patients and healthy controls was detected via dot blot assay (*n* = 25). (b) Total m^6^A levels in osteogenic differentiated or undifferentiated MC3T3‐E1 cells were detected by dot blot assay (*n* = 3). (c–h) Expression of METTL3, METTL14, FTO, ALKBH5, WTAP, and RBM15 in the serum samples of OP patients and healthy controls were evaluated by qPCR (*n* = 25). (i) Expression of ALKBH5 in differentiated or undifferentiated MC3T3‐E1 cells was evaluated by qPCR (*n* = 3). Student’s *t* test was used for statistical analysis (a–i). Data are expressed as mean ± SD. All *n* values represent the biological replicates. ^∗∗^ *p* < 0.01, ^∗∗∗^ *p* < 0.001.

**Figure:**
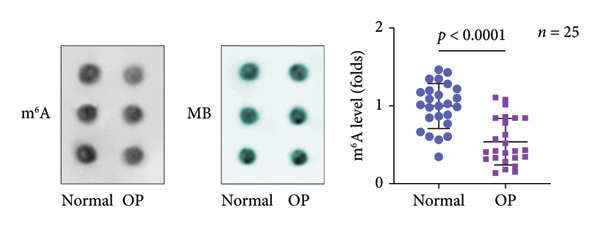
(a)

**Figure:**
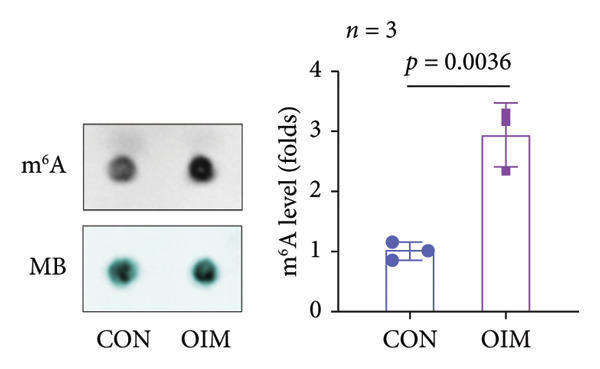
(b)

**Figure:**
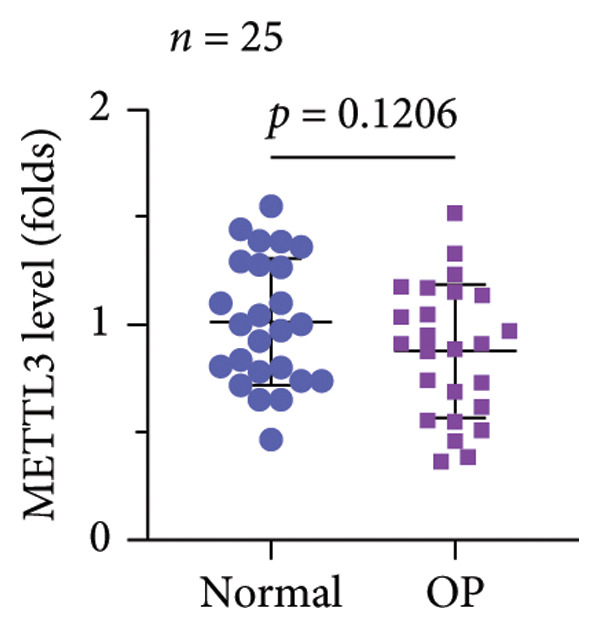
(c)

**Figure:**
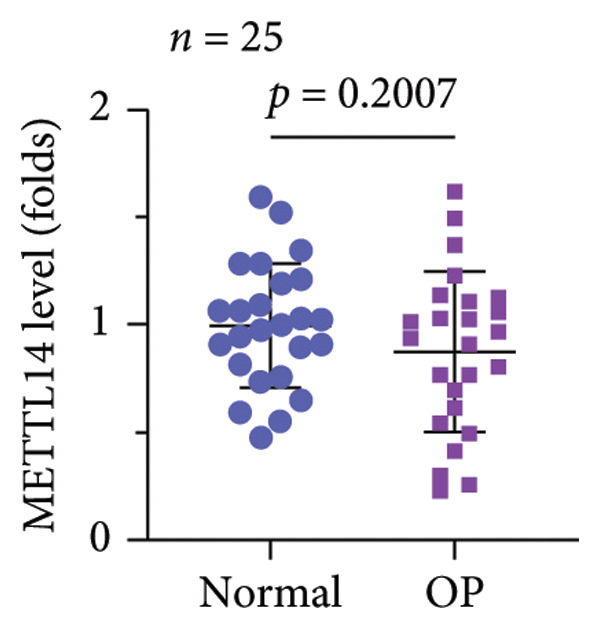
(d)

**Figure:**
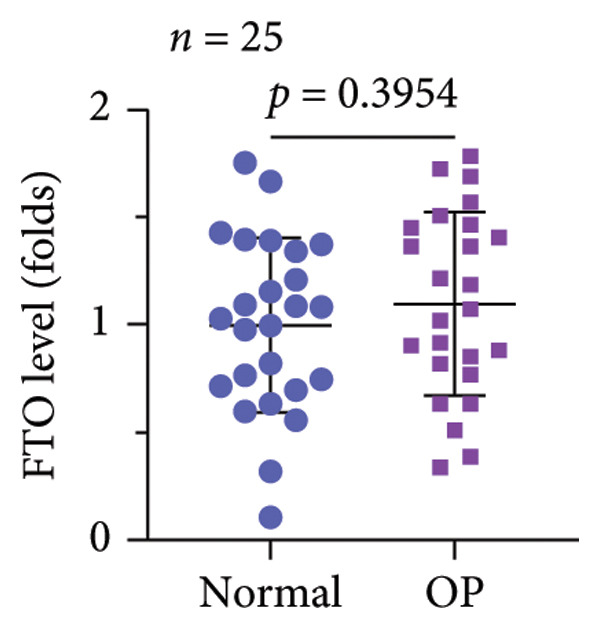
(e)

**Figure:**
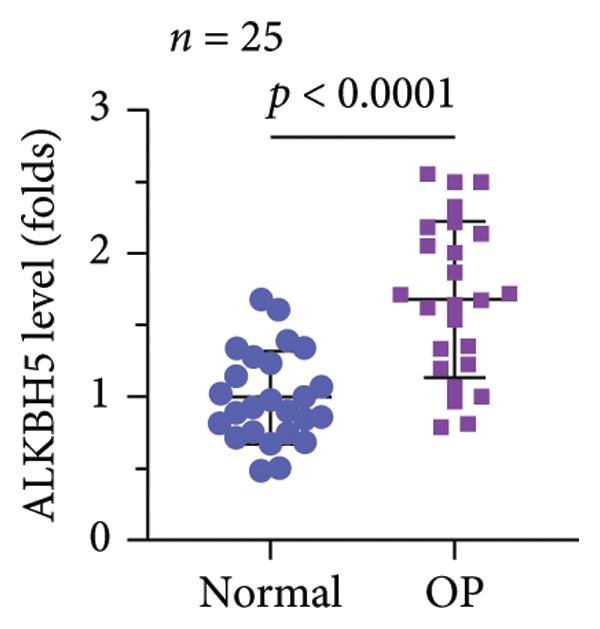
(f)

**Figure:**
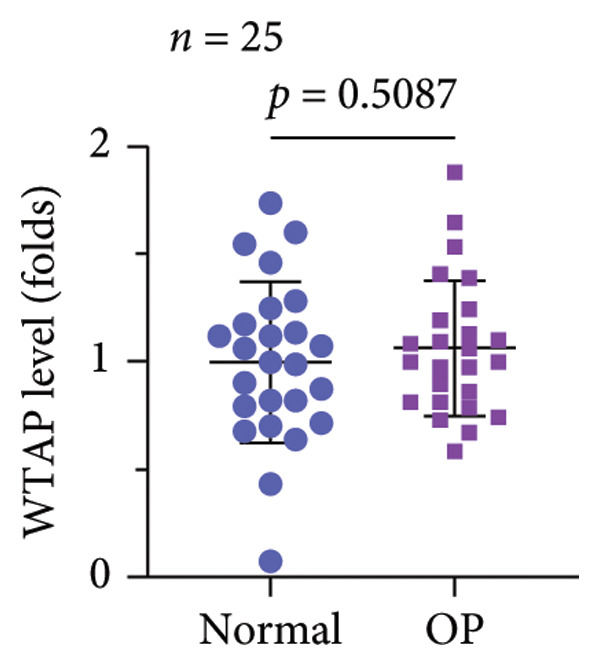
(g)

**Figure:**
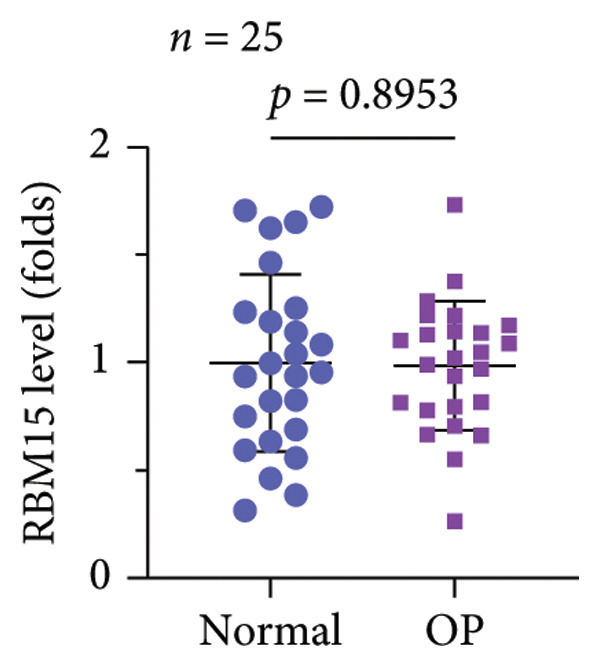
(h)

**Figure:**
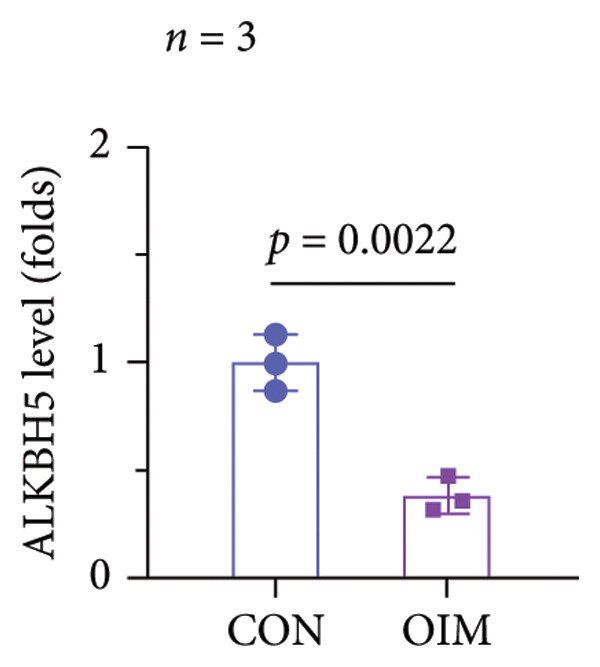
(i)

### 3.3. ALKBH5 Overexpression Inhibited the Osteogenic Differentiation in MC3T3‐E1 Cells

qPCR and western blot analyses confirmed significant upregulation of both ALKBH5 mRNA and protein levels post‐transfection (Figures 2(a), 2(b)). As expected, OIM enhanced cell viability, ALP activity, and ARS staining compared to undifferentiated controls. However, ALKBH5 overexpression markedly attenuated these osteogenic markers relative to the OIM + empty vector group (Figures 2(c), 2(d), 2(e)), indicating that ALKBH5 suppresses osteogenic differentiation. To corroborate these findings, parallel experiments were performed in hBMSCs, yielding consistent results (Supporting Figure 1).

Figure 2ALKBH5 overexpression inhibited the osteogenic differentiation in MC3T3‐E1 cells. (a–b) mRNA and protein levels of ALKBH5 after the transfection of ALKBH5 overexpression vectors were analyzed by qPCR and western blot (*n* = 3). (c) Cell viability in each group was evaluated by CCK‐8 assay (*n* = 3). (d) ALP activity was detected by an ALP activity kit (*n* = 3). (e) Calcium nodules were observed using an ARS kit (*n* = 3). Student’s *t* test (a and b) or one‐way ANOVA (c–e) was used for statistical analysis. Data are expressed as mean ± SD. All *n* values represent the biological replicates. ^∗∗^ *p* < 0.01, ^∗∗∗^ *p* < 0.001.

**Figure:**
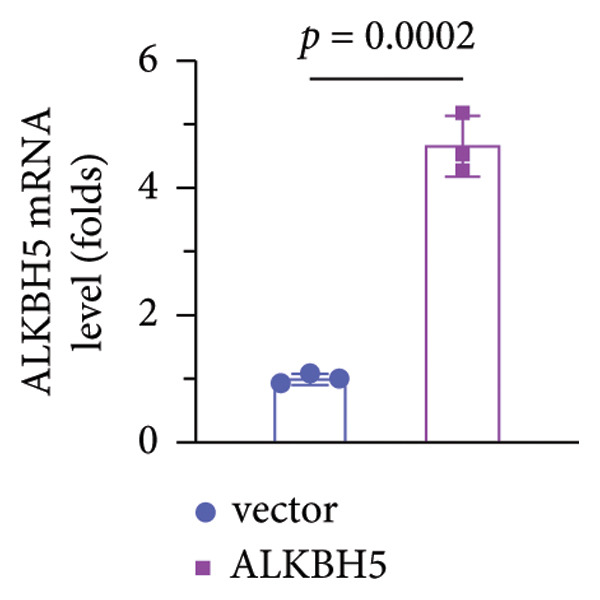
(a)

**Figure:**
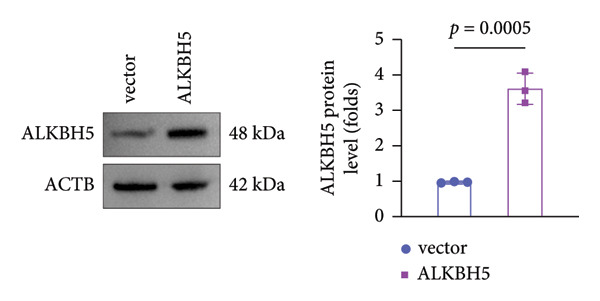
(b)

**Figure:**
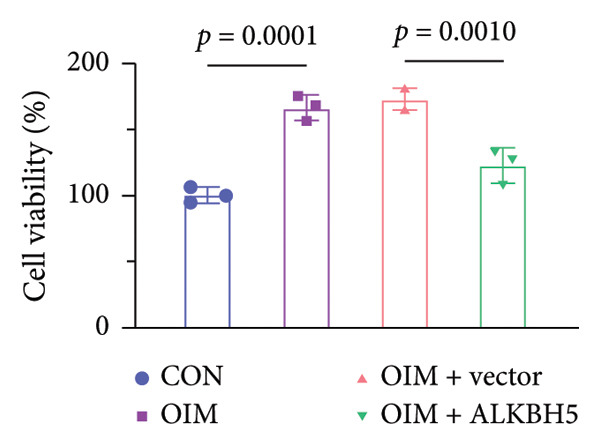
(c)

**Figure:**
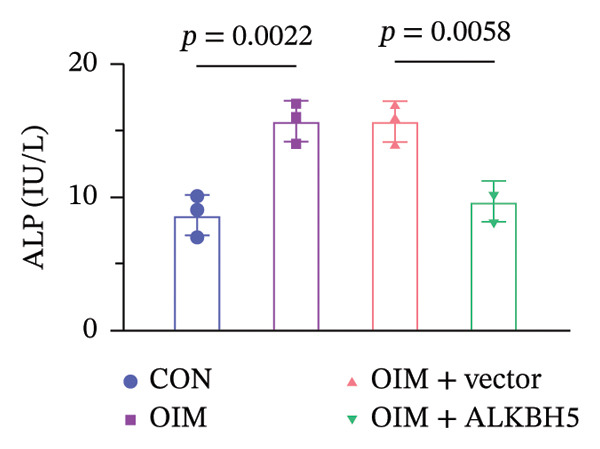
(d)

**Figure:**
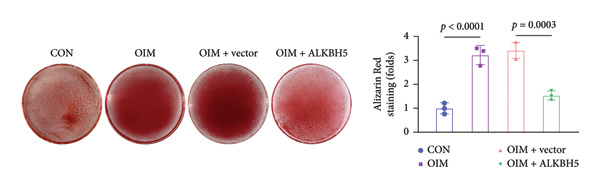
(e)

### 3.4. ELK1 Is a Key Downstream Effector of ALKBH5

To elucidate the molecular mechanisms of ALKBH5, candidate target genes were first predicted using GeneMANIA (Figure 3(a)). Pathway enrichment analysis of ALKBH5‐associated proteins was then performed via FunRich (Figure 3(b)). Among the candidates, ELK1—a member of the ETS family of transcription factors that binds specific DNA motifs to regulate proliferation, differentiation, and apoptosis [18]—was notably enriched in multiple osteogenic pathways, including IGF‐1, IFN‐γ, and mTOR signaling. Given its functional relevance, ELK1 was selected for further study. Using the SRAMP database, two putative m^6^A sites were identified in ELK1 mRNA (positions 675–679 [S1] and 1683–1687 [S2]; Figure 3(c)). qPCR showed that ALKBH5 overexpression reduced mature ELK1 mRNA levels without affecting its precursor (Figure 3(d)), suggesting post‐transcriptional regulation. MeRIP‐qPCR confirmed a decrease in ELK1 m^6^A methylation upon ALKBH5 overexpression (Figure 3(e)). Luciferase reporter assays revealed that the ALKBH5 specifically suppressed activity of the wild‐type S1 construct, but not S2 (Figure 3(f)), identifying S1 as the functional m^6^A site. Furthermore, Actinomycin D chase experiments demonstrated accelerated decay of ELK1 mRNA in ALKBH5‐overexpressing cells (Figure 3(g)), indicating reduced transcript stability.

Figure 3ELK1 is a key downstream effector of ALKBH5. (a) The genes that can be potentially regulated by ALKBH5 were screened using GENEMANIA database. (b) Enrichment analysis was performed using these genes and FunRich tool. (c) The potential m^6^A modified sites were predicted by SRAMP software. (d) The expression of the ELK1 precursor and maturate after ALKBH5 overexpression was detected by qPCR (*n* = 3). (e) The m^6^A level of ELK1 after ALKBH5 overexpression was evaluated by MeRIP assay (*n* = 3). (f) The binding relationship between ALKBH5 and ELK1 at Sites 1 and 2 was evaluated by luciferase reporter assay (*n* = 3). (g) ELK1 mRNA stability was analyzed after ALKBH5 overexpression (*n* = 3). Student’s *t* test (d–g) was used for statistical analysis. Data are expressed as mean ± SD. All *n* values represent the biological replicates.

**Figure:**
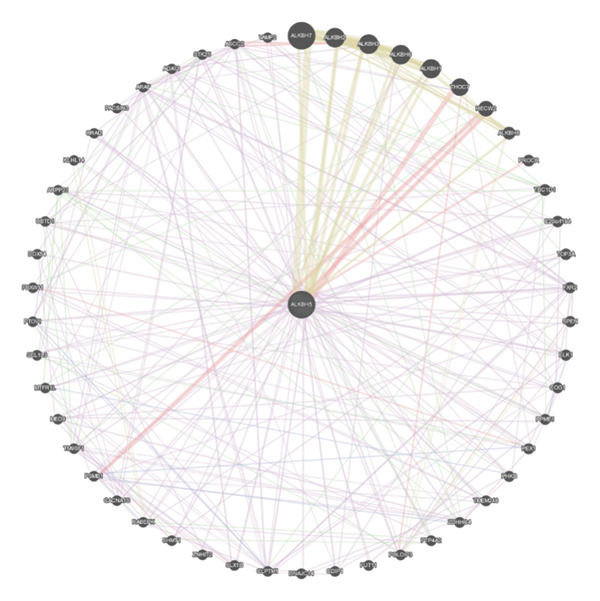
(a)

**Figure:**
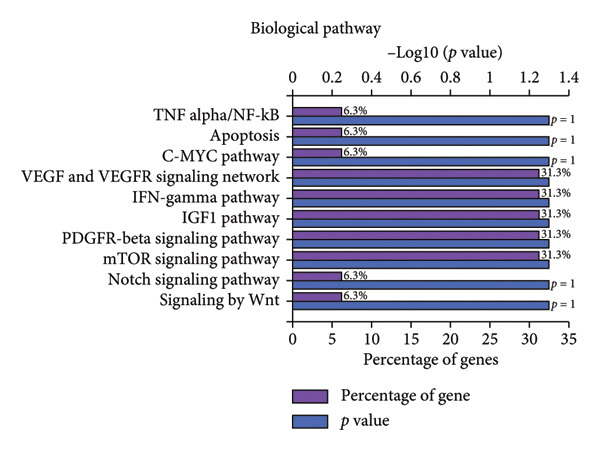
(b)

**Figure:**
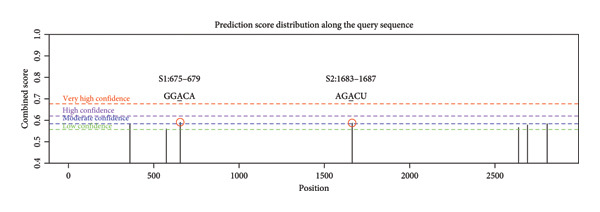
(c)

**Figure:**
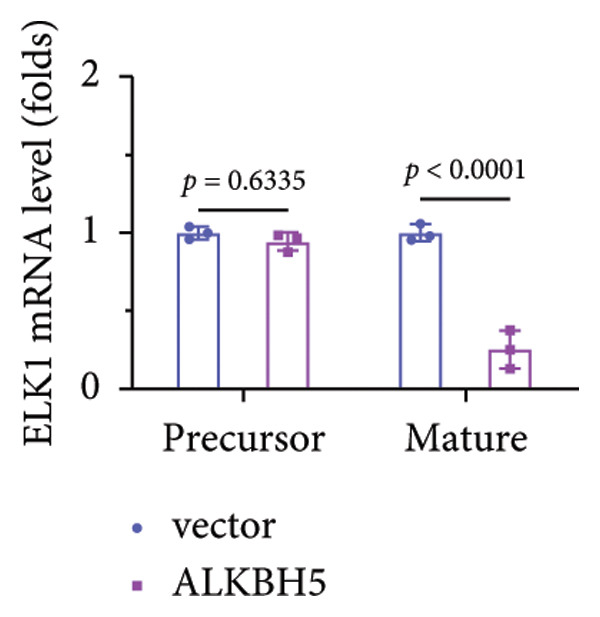
(d)

**Figure:**
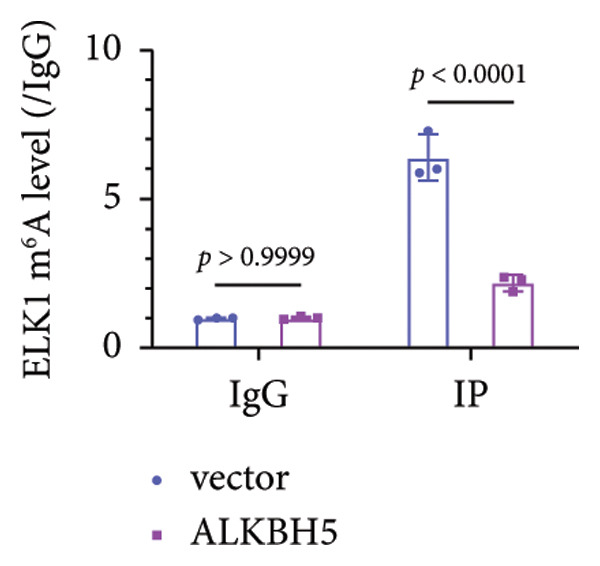
(e)

**Figure:**
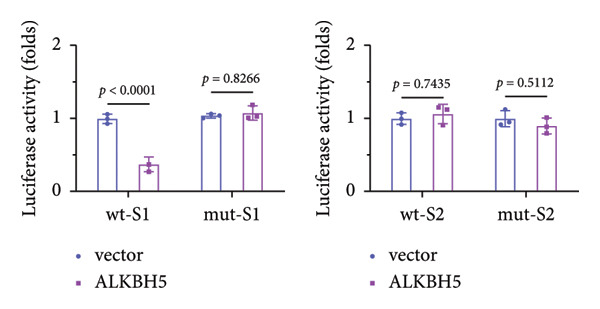
(f)

**Figure:**
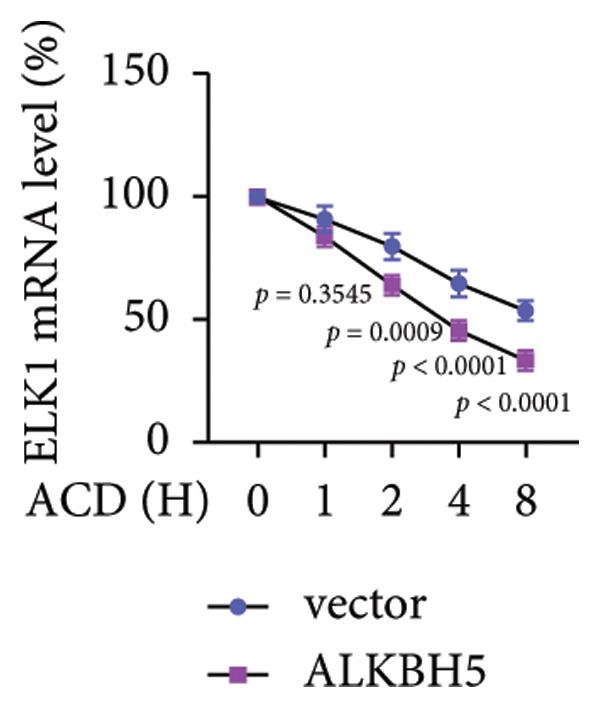
(g)

### 3.5. YTHDF2 Recognized ALKBH5‐Regulated ELK1 Demethylation

To investigate how m^6^A modification influences ELK1 expression, we transfected MC3T3‐E1 cells with overexpression vectors of m^6^A “readers.” The results confirmed the upregulation of these genes post‐transfection (Figures 4(a), 4(b)). Additionally, only YTHDF2 overexpression downregulated ELK1 expression, while YTHDC1, YTHDC2, YTHDF1, and YTHDF3 had no effect (Figure 4(c)), indicating YTHDF2 as a specific m^6^A “reader” for ALKBH5‐mediated m^6^A demethylation. RIP assays demonstrated direct binding between YTHDF2 and ELK1 (Figure 4(d)), further supported by luciferase assays (Figure 4(e)). After transfecting the si‐ALKBH5 vector into MC3T3‐E1 cells, the expressions of ALKBH5 were decreased (Figures 4(f), 4(g)). Besides, silencing of ALKBH5 upregulated the mRNA level of ELK1, while silencing of YTHDF2 restored the elevation induced by ALKBH5 (Figure 4(h)). These findings highlighted YTHDF2 as a key mediator of ALKBH5‐regulated ELK1 expression through m^6^A‐dependent mechanisms.

Figure 4YTHDF2 recognized ALKBH5‐regulated ELK1 demethylation. (a–b) The mRNA and protein levels of YTHDC1, YTHDC2, YTHDF1, YTHDF2, and YTHDF3 in MC3T3‐E1 cells after YTHDC1, YTHDC2, YTHDF1, YTHDF2, and YTHDF3 overexpression were assessed by qPCR and western blot (*n* = 3). (c) The expression of ELK1 was detected after the overexpression of YTHDC1, YTHDC2, YTHDF1, YTHDF2, and YTHDF3 (*n* = 3). (d) RIP assay was performed to assess the binding between YTHDF2 and ELK1 (*n* = 3). (e) The luciferase assay was used to verify the binding YTHDF2 and ELK1 (*n* = 3). (f‐g) The mRNA and protein levels of ALKBH5 in MC3T3‐E1 cells were analyzed by qPCR and western blot after the transfection of si‐NC and si‐ALKBH5 (*n* = 3). (h) ELK1 expression in each group was detected by qPCR (*n* = 3). Student’s *t* test (a–g) or one‐way ANOVA (h) was used for statistical analysis. Data are expressed as mean ± SD. All *n* values represent the biological replicates.

**Figure:**
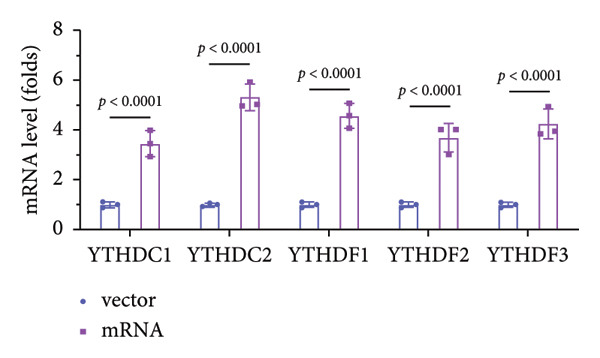
(a)

**Figure:**
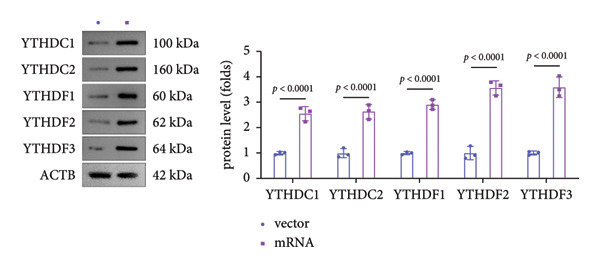
(b)

**Figure:**
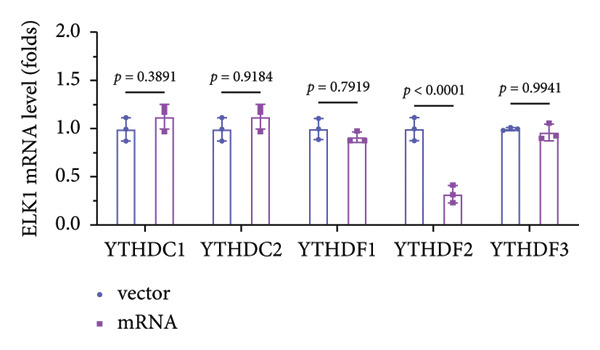
(c)

**Figure:**
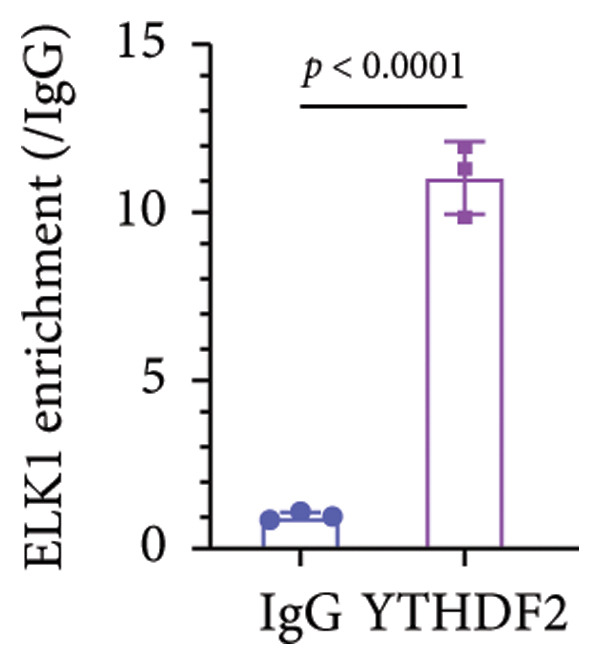
(d)

**Figure:**
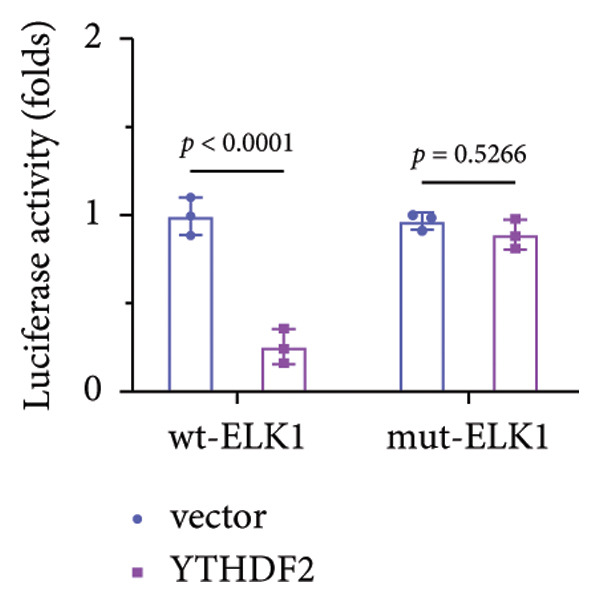
(e)

**Figure:**
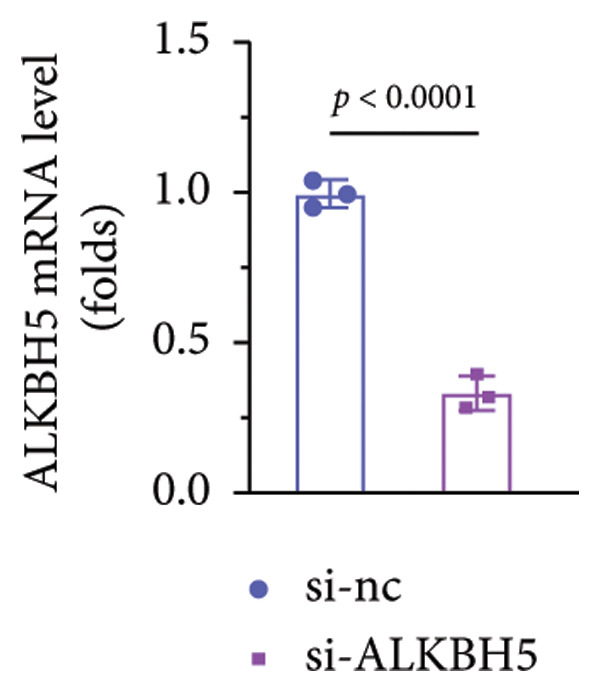
(f)

**Figure:**
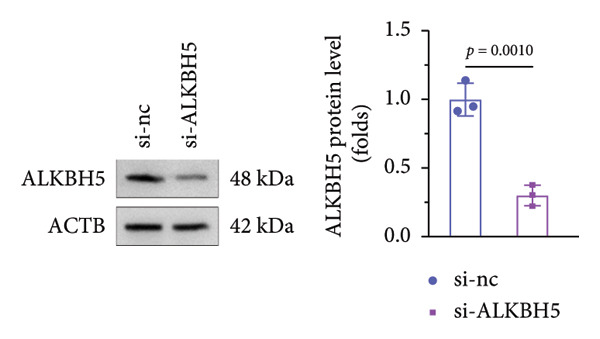
(g)

**Figure:**
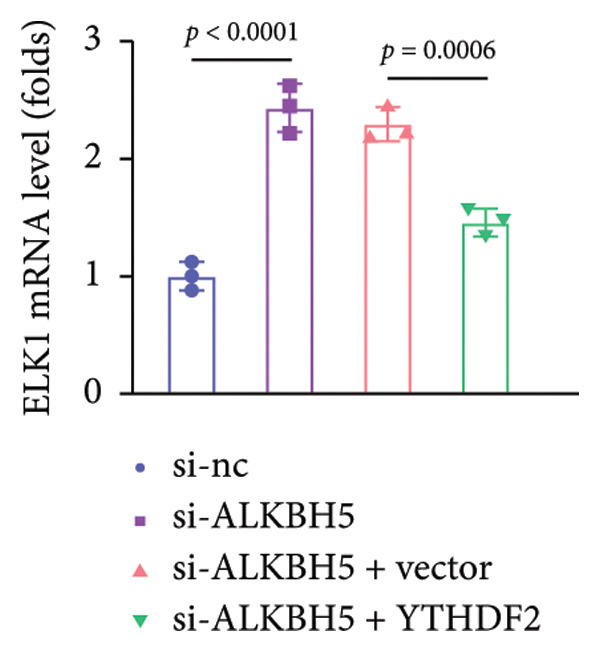
(h)

### 3.6. ELK1 Overexpression Counteracted the Suppressive Effect of ALKBH5 Overexpression on Osteogenic Differentiation

Rescue experiments were conducted to assess whether ELK1 overexpression could counteract the suppressive effects of ALKBH5 on osteogenic differentiation. Transfection with an ELK1 overexpression vector successfully elevated both mRNA and protein levels (Figures 5(a), 5(b)). Functional assays revealed that ELK1 restoration reversed ALKBH5‐mediated inhibition of cell viability, ALP activity, and mineralized nodule formation in MC3T3‐E1 cells (Figures 5(c), 5(d), 5(e)).

Figure 5Overexpression of ELK1 reversed the inhibition of osteogenic differentiation induced by ALKBH5 overexpression. (a and b) mRNA and protein levels of ELK1 after ELK1 overexpression were analyzed by qPCR and western blot (*n* = 3). (c) Cell viability in each group was evaluated by CCK‐8 assay (*n* = 3). (d) ALP activity in each group was detected by an ALP activity kit (*n* = 3). (e) Calcium nodules in each group were observed using ARS staining (*n* = 3). Student’s *t* test (a and b) or one‐way ANOVA (c–e) was used for statistical analysis. Data are expressed as mean ± SD. All *n* values represent the biological replicates.

**Figure:**
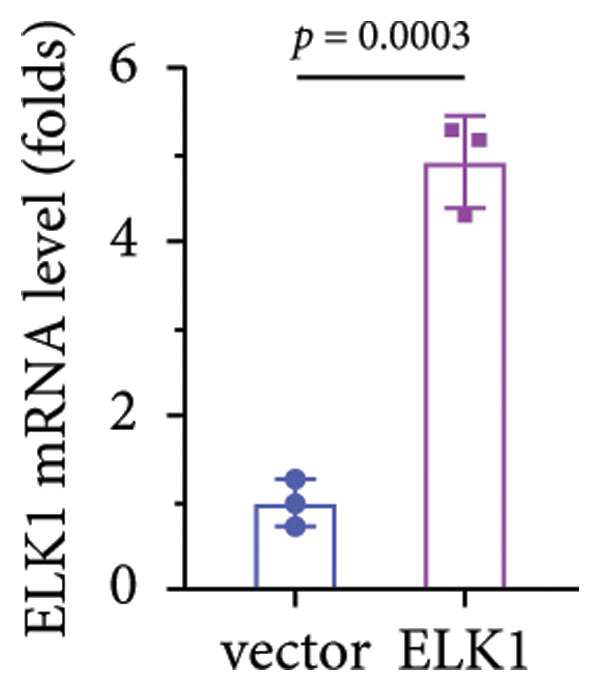
(a)

**Figure:**
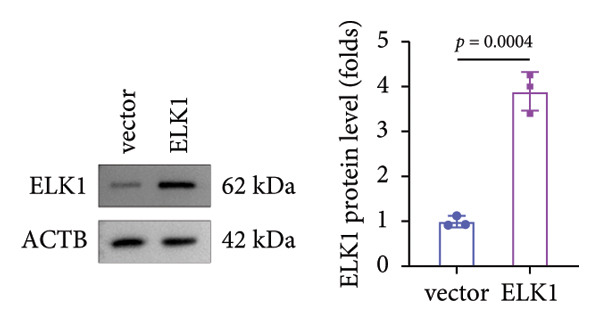
(b)

**Figure:**
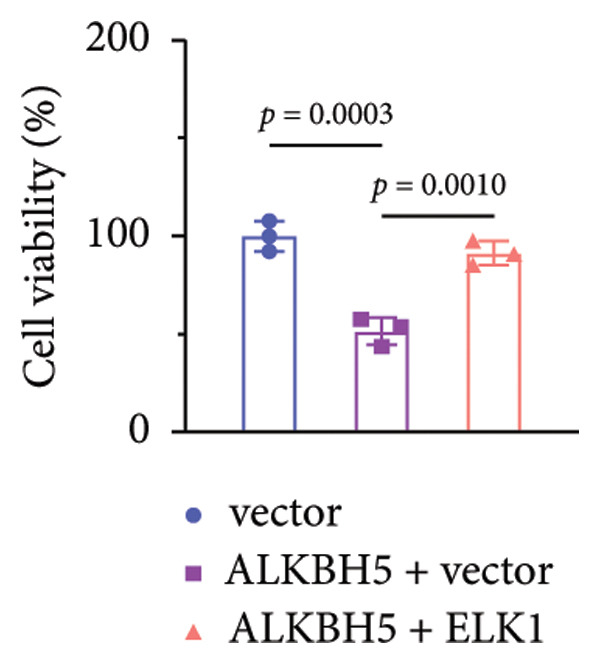
(c)

**Figure:**
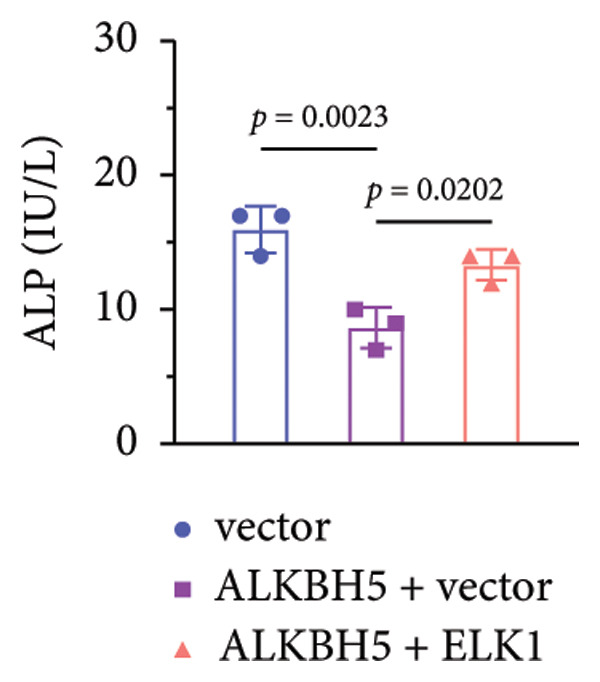
(d)

**Figure:**
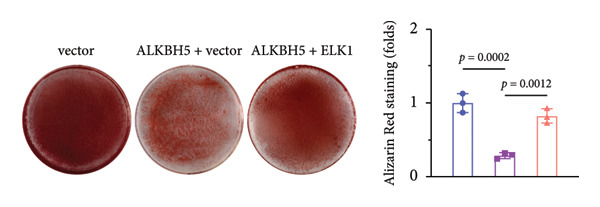
(e)

### 3.7. ELK1 Knockdown Reversed the Promoted Osteogenic Differentiation Induced by YTHDF2 Inhibition

qPCR and western blot confirmed efficient knockdown of YTHDF2 and ELK1 (Figures 6(a), 6(b)). Relative to the si‐NC group, YTHDF2 silencing enhanced MC3T3‐E1 cell viability, ALP activity, and ARS staining area. Conversely, cosilencing of ELK1 in YTHDF2‐deficient cells (si‐YTHDF2 + si‐ELK1) significantly reversed these effects relative to the si‐YTHDF2 + si‐NC group (Figures 6(c), 6(d), 6(e)).

Figure 6ELK1 knockdown reversed the promoted osteogenic differentiation induced by YTHDF2 inhibition in MC3T3‐E1 cells. (a and b) mRNA and protein levels of ALKBH5 and EKL1 after transfection of ALKBH5 and EKL1 knockdown vectors were analyzed by qPCR and western blot (*n* = 3). (c) Cell viability in each group was evaluated by CCK‐8 assay (*n* = 3). (d) ALP activity was detected by an ALP activity kit (*n* = 3). (e) Calcium nodules were observed using an ARS kit (*n* = 3). Student’s *t* test (a and b) or one‐way ANOVA (c–e) was used for statistical analysis. Data are expressed as mean ± SD. All *n* values represent the biological replicates.

**Figure:**
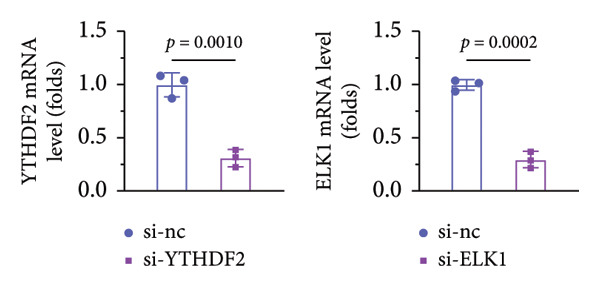
(a)

**Figure:**
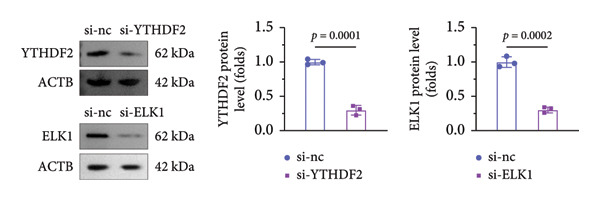
(b)

**Figure:**
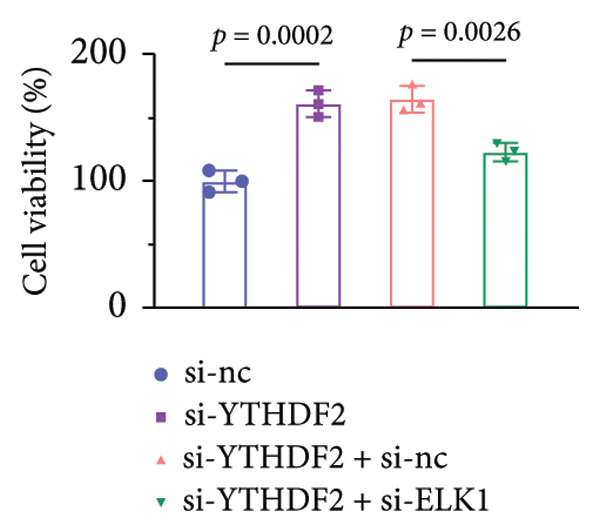
(c)

**Figure:**
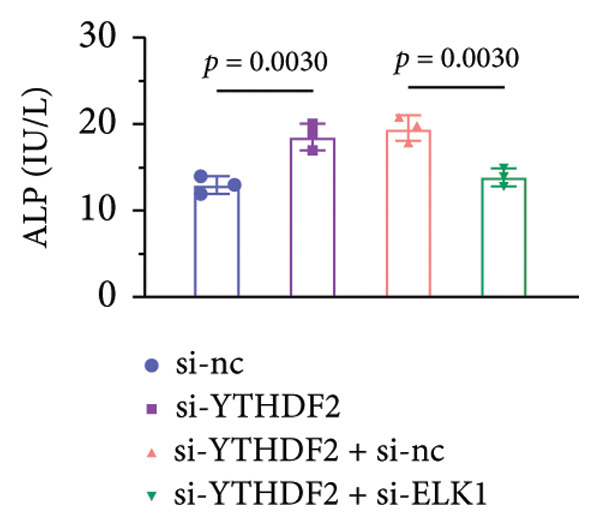
(d)

**Figure:**
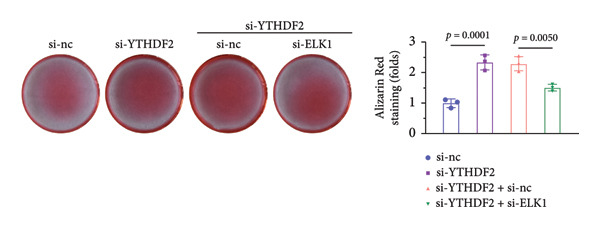
(e)

### 3.8. ALKBH5 Knockdown Attenuated OVX‐Induced Bone Loss and Improved Bone Strength

Finally, an OVX‐induced OP mouse model was established. Compared with sham controls, OVX mice exhibited elevated ALKBH5 mRNA and protein levels in femoral tissue; this upregulation was effectively reversed by sh‐ALKBH5 (Figures 7(a), 7(b)). Concurrently, OVX increased YTHDF2 but decreased ELK1 expression, whereas ALKBH5 knockdown restored both to near‐basal levels (Figures 7(c), 7(d)). Micro‐CT analysis revealed severe trabecular bone loss in the OVX group versus Sham, characterized by reduced BMD, BV/TV, Tb.N, and Tb.Th, along with increased Tb.Sp and TBPf. In contrast, ALKBH5 silencing significantly ameliorated these structural deficits compared to the OVX + sh‐NC group (Figures 7(e), 7(f), 7(g), 7(h), 7(i), 7(j), 7(k)). Histologically, H & E staining showed disorganized, sparse trabeculae in OVX mice, while sh‐ALKBH5 treatment improved trabecular thickness and continuity (Figure 7(l)). TRAP staining demonstrated a marked increase in osteoclast numbers in OVX mice, which was attenuated by ALKBH5 knockdown (Figures 7(m), 7(n)). Finally, biomechanical testing confirmed that OVX impaired bone strength—evidenced by reduced elastic modulus and stiffness (K)—and that ALKBH5 inhibition partially rescued these mechanical properties (Figures 7(o), 7(p)).

Figure 7ALKBH5 knockdown attenuated OVX‐induced bone loss and improved bone strength. (a and b) ALKBH5 mRNA and protein levels in each group were analyzed by qPCR and western blot (*n* = 6). mRNA levels of (c) YTHDF2, and (d) ELK1 in each group were analyzed by qPCR (*n* = 6). (e) Three‐dimensional CT image of femur (scale bar = 5 mm) (*n* = 6); quantitative data of (f) BMD, (g) BV/TV, (h) Tb.N, (i) Tb.Sp, (j) Tb.Th, and (k) TBPf of femur tissues in mice (*n* = 6). (l) HE staining was performed to show histopathological changes of femur tissues in each group (scale bar = 200 μm) (*n* = 6). (m and n) TRAP staining and quantitative analysis of TRAP‐positive cell numbers in each group (scale bar = 200 μm) (*n* = 6). (o and p) Mechanical properties of femora including elasticity modulus and stiffness constant K (*n* = 6). One‐way ANOVA (a–p) was used for statistical analysis. Data are expressed as mean ± SD. All *n* values represent the biological replicates.

**Figure:**
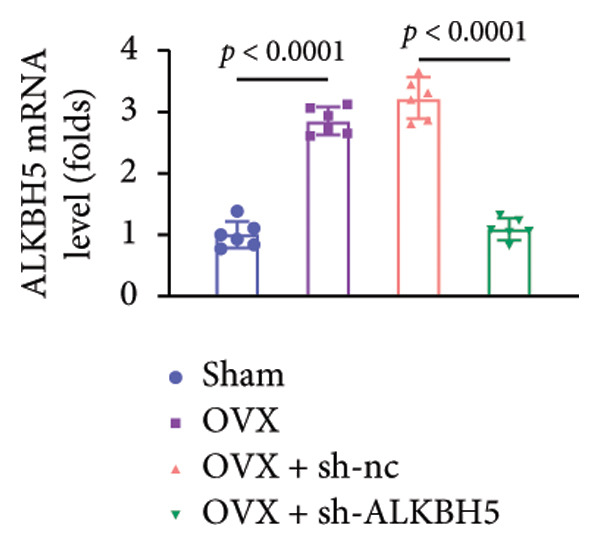
(a)

**Figure:**
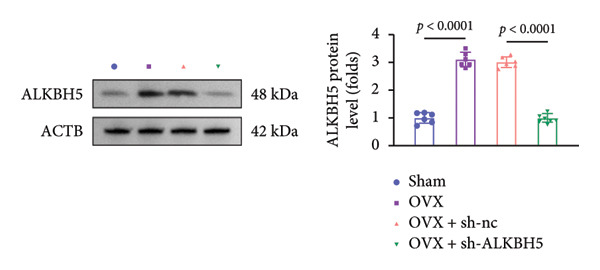
(b)

**Figure:**
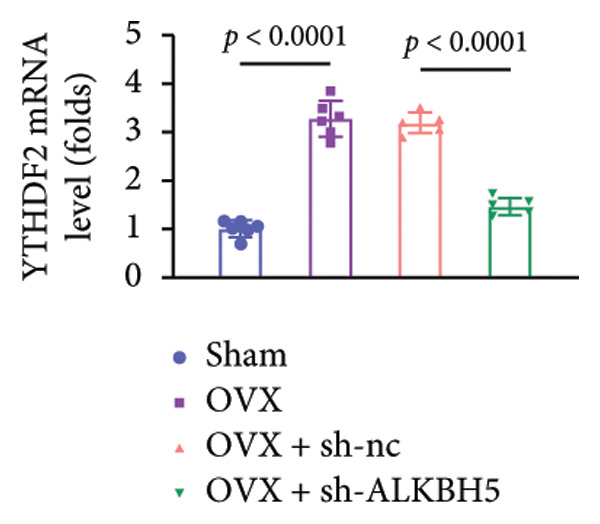
(c)

**Figure:**
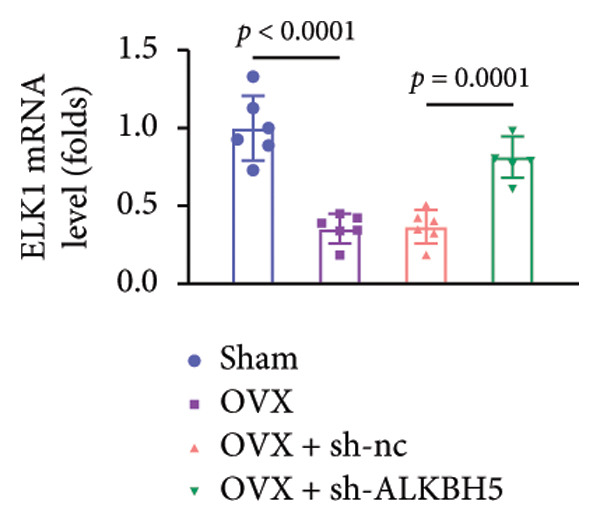
(d)

**Figure:**
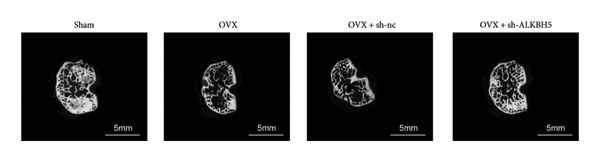
(e)

**Figure:**
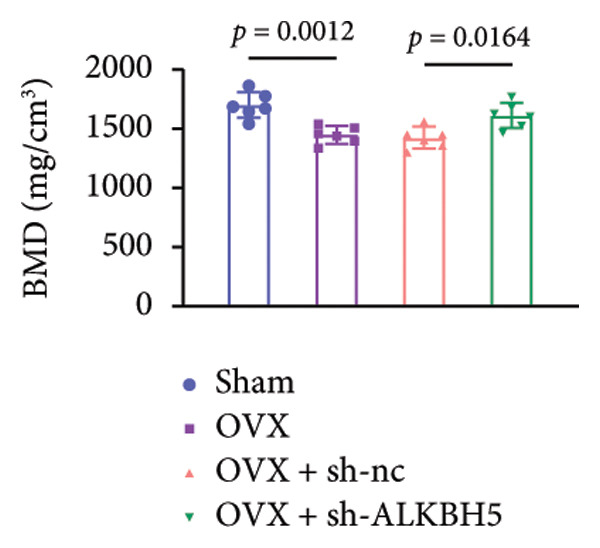
(f)

**Figure:**
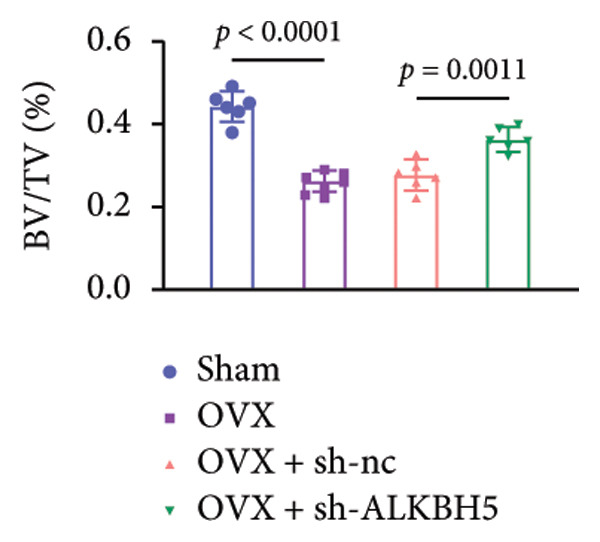
(g)

**Figure:**
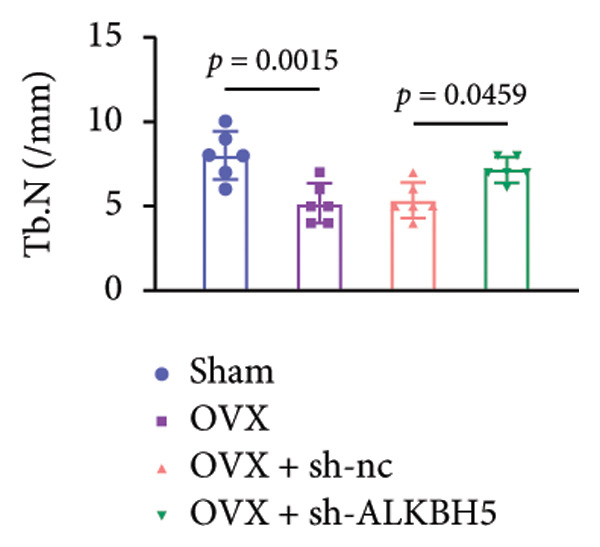
(h)

**Figure:**
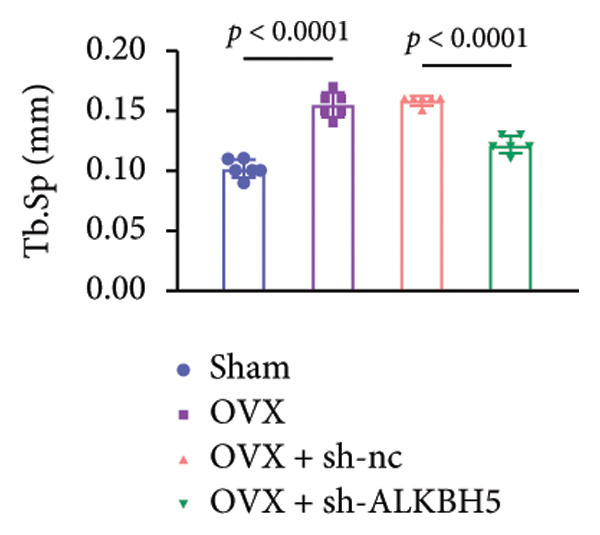
(i)

**Figure:**
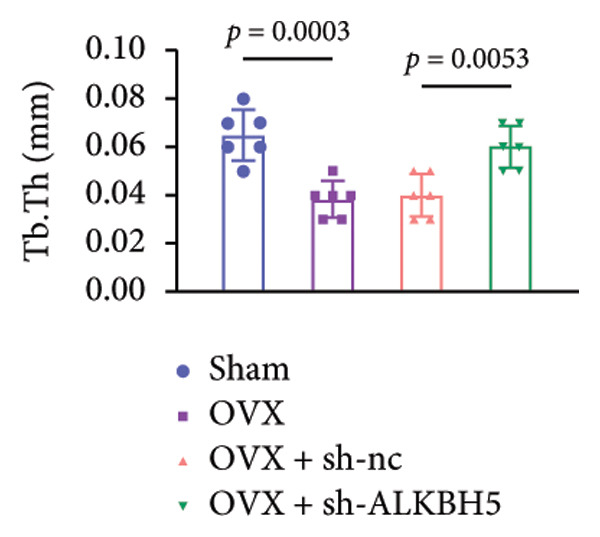
(j)

**Figure:**
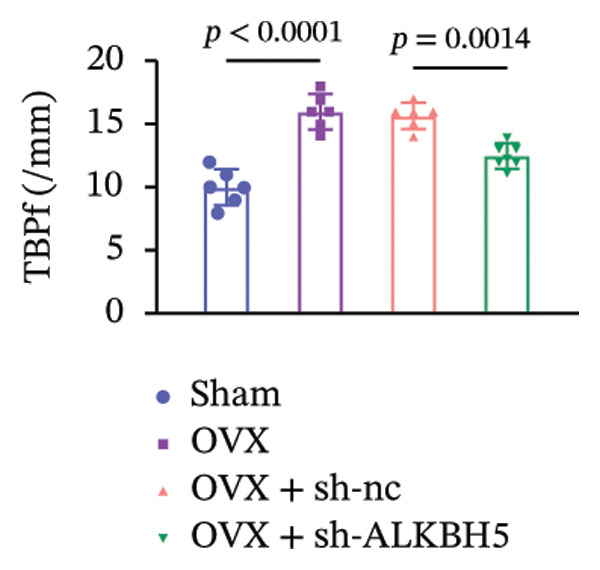
(k)

**Figure:**
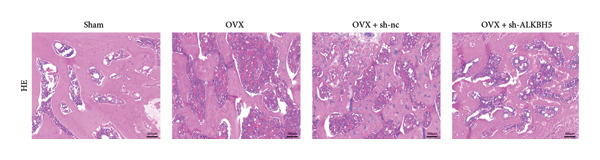
(l)

**Figure:**
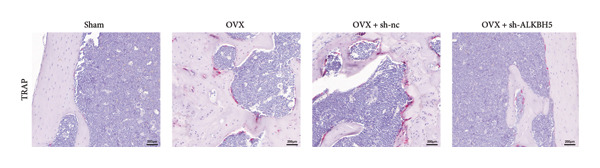
(m)

**Figure:**
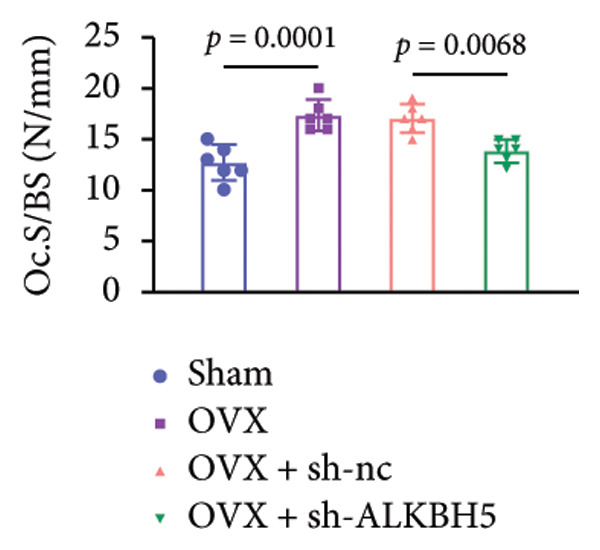
(n)

**Figure:**
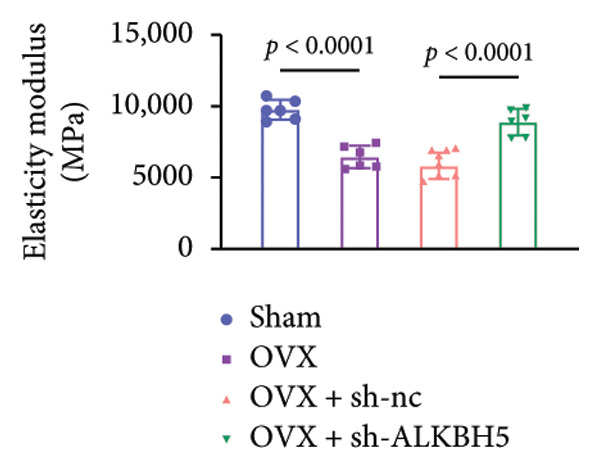
(o)

**Figure:**
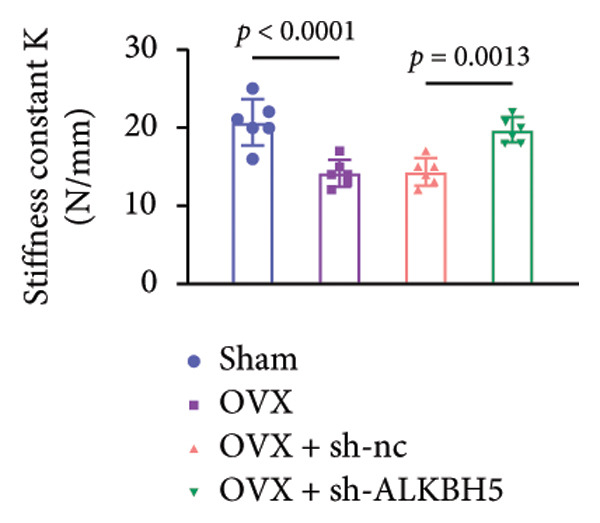
(p)

### 3.9. ELK1 Knockdown Reversed the Reduced Bone Loss and the Improved Bone Strength Induced by YTHDF2 Knockdown in OVX Mice

To dissect the functional interplay between YTHDF2 and ELK1 in bone homeostasis, shRNA vectors targeting YTHDF2 and/or ELK1 were delivered into OVX mice. qPCR and western blot confirmed efficient knockdown of both targets relative to the sh‐NC control (Figures 8(a), 8(b)). Micro‐CT analysis showed that YTHDF2 silencing markedly enhanced trabecular bone mass compared to sh‐NC, whereas coknockdown of ELK1 attenuated this anabolic effect relative to sh‐YTHDF2 + sh‐NC (Figure 8(c)). Quantitatively, sh‐YTHDF2 increased BMD, BV/TV, Tb.N, and Tb.Th while decreasing Tb.Sp and TBPf; these improvements were reversed upon ELK1 depletion (Figures 8(d), 8(e), 8(f), 8(g), 8(h), 8(i)). Histologically, H & E staining revealed that YTHDF2 knockdown promoted thicker, more continuous trabeculae versus sh‐NC, but this structural benefit was abolished by concurrent ELK1 silencing (Figure 8(j)). Consistently, TRAP staining demonstrated fewer osteoclasts in the sh‐YTHDF2 group compared to sh‐NC, whereas the sh‐YTHDF2 + sh‐ELK1 group exhibited a significant rebound in TRAP^+^ cell numbers, indicating restored osteoclast activity (Figures 8(k), 8(l)). Finally, biomechanical testing showed that YTHDF2 deficiency enhanced bone strength—evidenced by elevated elastic modulus and stiffness (K)—and that ELK1 coknockdown abrogated this mechanical advantage (Figures 8(m), 8(n)).

Figure 8ELK1 knockdown reversed the reduced bone loss and the improved bone strength induced by YTHDF2 knockdown in OVX mice. (a and b) The mRNA and protein levels of YTHDF2 and ELK1 in femur tissues were analyzed by qPCR and western blot after YTHDF2 and ELK1 knockdown (*n* = 6). (c) Three‐dimensional CT image of femur (scale bar = 5 mm) (*n* = 6); quantitative data of (d) BMD, (e) BV/TV, (f) Tb.N, (g) Tb.Sp, (h) Tb.Th, and (i) TBPf of femur tissues in mice (*n* = 6). (j) HE staining was performed to show histopathological changes of femur tissues in each group (scale bar = 200 μm) (*n* = 6). (k and l) TRAP staining and quantitative analysis of TRAP‐positive cell numbers in each group (scale bar = 200 μm) (*n* = 6). (m and n) Mechanical properties of femora including elasticity modulus and stiffness constant K (*n* = 6). One‐way ANOVA (a–n) was used for statistical analysis. Data are expressed as mean ± SD. All *n* values represent the biological replicates.

**Figure:**
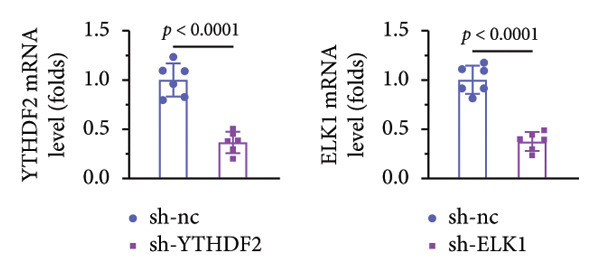
(a)

**Figure:**
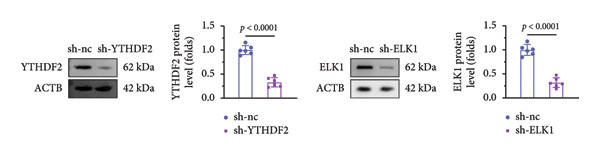
(b)

**Figure:**
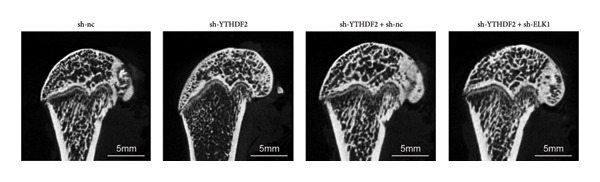
(c)

**Figure:**
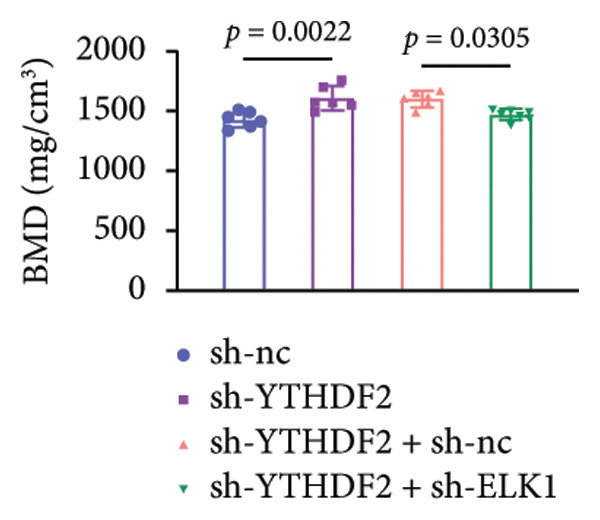
(d)

**Figure:**
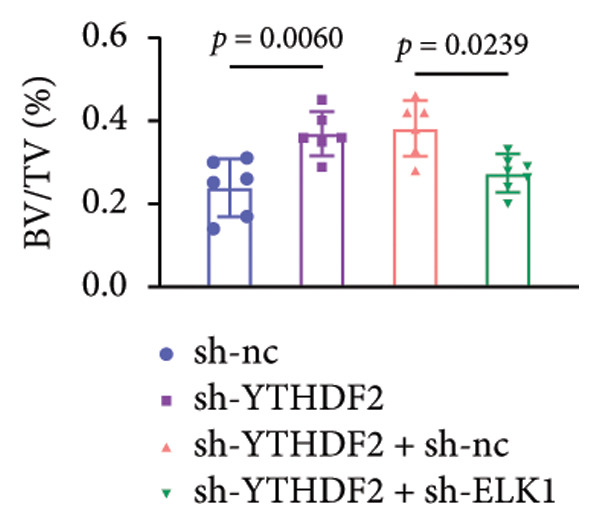
(e)

**Figure:**
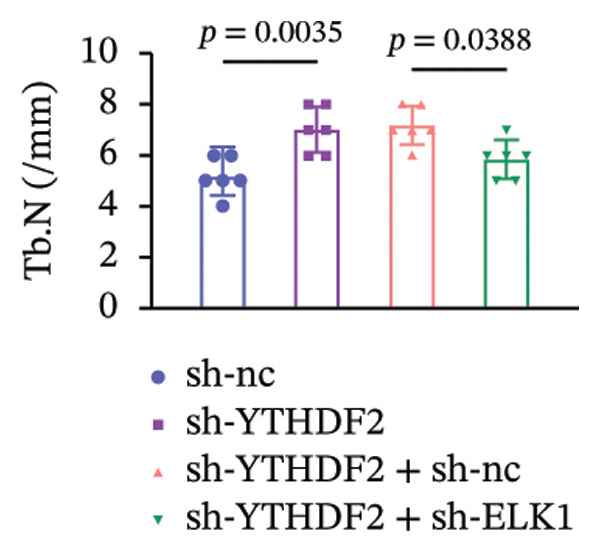
(f)

**Figure:**
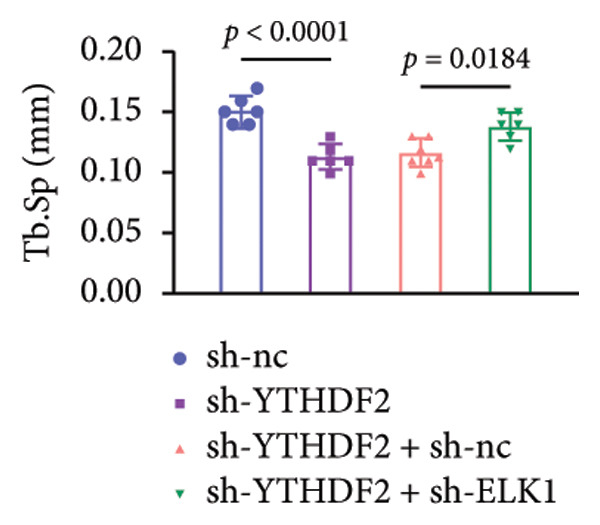
(g)

**Figure:**
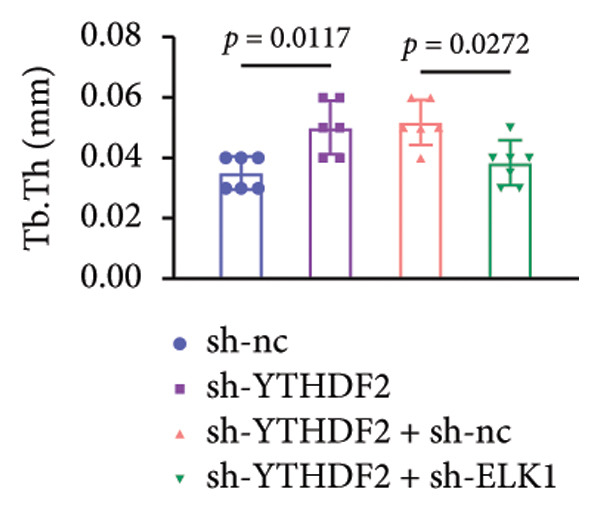
(h)

**Figure:**
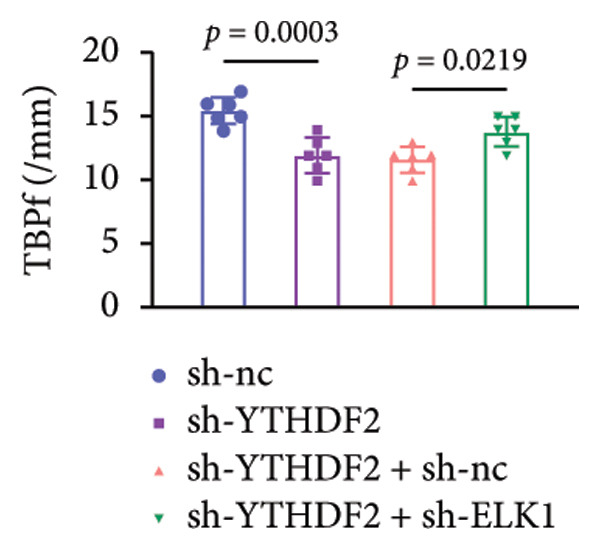
(i)

**Figure:**
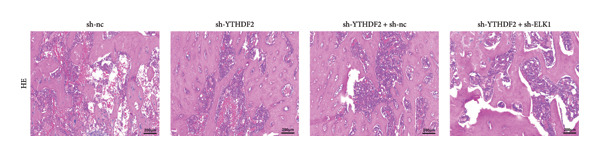
(j)

**Figure:**
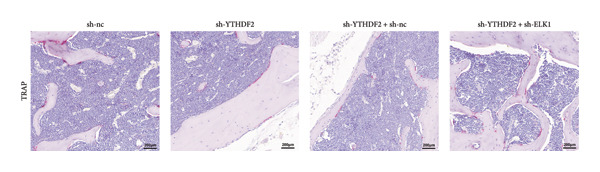
(k)

**Figure:**
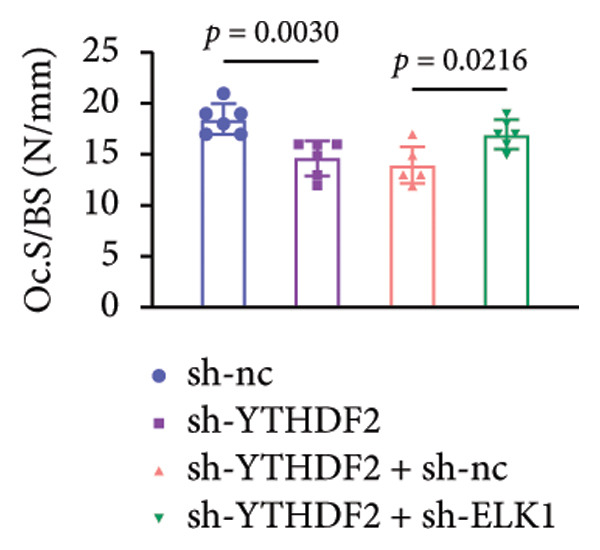
(l)

**Figure:**
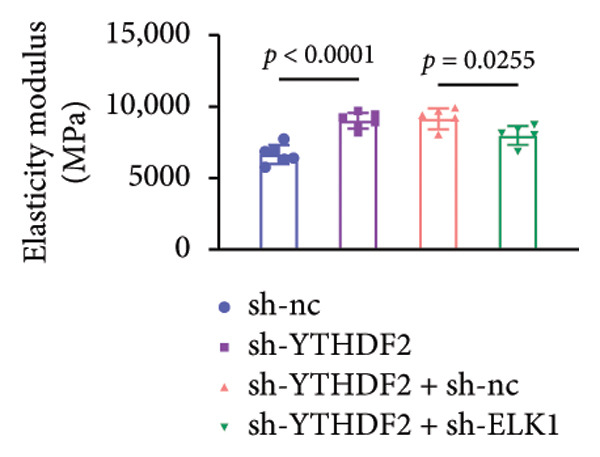
(m)

**Figure:**
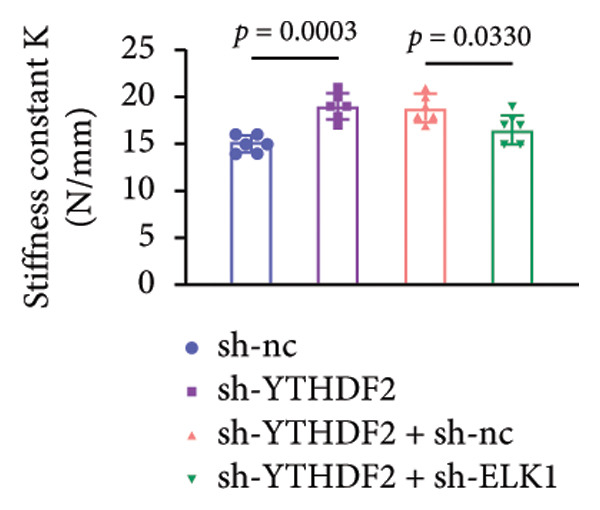
(n)

## 4. Discussion

In the present study, we profiled the expression of multiple m^6^A writer and eraser genes in serum from OP patients and in MC3T3‐E1 pre‐osteoblasts, identifying ALKBH5 as a candidate regulator. Functional assays demonstrated that ALKBH5 suppresses osteogenic differentiation. Consistently, Li et al. [9] demonstrate that ALKBH5 inhibits osteogenesis in mesenchymal stem cells (MSCs) *in vitro*, and Huang et al. [8] showed that ALKBH5 inhibition alleviates etoposide‐induced senescence and promotes osteogenic differentiation. Notably, however, ALKBH5 appears to exert context‐dependent effects: In contrast to these observations, one study reported that ALKBH5 enhances osteoblast differentiation in MSCs [19]. Besides, Weng et al. [20] reveal that an abnormal reduction in ALKBH5 expression in macrophages leads to impaired polarization and suppresses osteoblast differentiation in diabetic peri‐implantitis. These differences might arise from the differences in experimental conditions, cell types, or disease contexts, leading to varying roles of ALKBH5 in osteogenesis.

ELK1 is a key regulator of gene expression and essential cellular functions such as differentiation and proliferation. Accumulating evidence implicates ELK1 in OP, particularly in modulating bone remodeling and the balance between osteoblast and osteoclast activity [11, 21]. In this study, we found that ALKBH5 inhibited osteogenic difference by inhibiting the m^6^A methylation of ELK1, a key downstream effector. ALKBH5 could bind with 675–679 bp of ELK1 mRNA and reduce its stability. Besides, the overexpression of ELK1 promoted osteogenic differentiation. This study is the first to reveal that ELK1 regulates osteogenic differentiation through m^6^A modification. Consistent with our results, ELK1 has been shown to promote osteoblast differentiation and inhibit apoptosis in MC3T3‐E1 cells [11]. Li et al. [2] report that ELK1 enhances osteogenesis via the MAPK/ERK pathway, while Liu et al. [12] demonstrate that exosomal HCP5 promotes osteogenic differentiation through the miR‐24‐3p/HO‐1/p38/ELK1 axis.

The function of m^6^A modification depends on the recognition by specific “readers,” which bind to and influence the stability or translation of target RNAs. In this study, only YTHDF2 was found to inhibit the expression of ELK1. Rescue experiments confirmed our hypothesis, suggesting that YTHDF2 acts as a “reader” of ALKBH5 in OP. The role of ALKBH5/YTHDF2‐mediated m^6^A modification in OP has not been previously explored. Notably, ALKBH5 has been reported to suppress osteogenic differentiation in etoposide‐induced senescent cells by removing m^6^A modifications from VDAC3 mRNA, which is then recognized by m^6^A reader YTHDF1 [8]. Beyond bone biology, the ALKBH5–YTHDF2 axis also contributes to pathogenesis in other contexts. For example, Shao et al. [22] reveal that the ALKBH5‐mediated demethylation of circAFF2 promotes its YTHDF2‐dependent degradation, thereby modulating radiosensitivity in colorectal cancer. Similarly, Xiao et al. [23] discover that in rheumatoid arthritis, ALKBH5 reduces NLRP3 mRNA stability through m^6^A modification recognized by YTHDC2, leading to suppressed NLRP3 expression. These findings highlighted the diverse and context‐dependent roles of ALKBH5/YTHDF2‐mediated m^6^A modification across different biological processes and diseases.

In summary, our study shows that ALKBH5 is upregulated in OP and inhibits osteogenic differentiation. Mechanistically, ALKBH5 directly targets ELK1—a key downstream effector—and downregulates its expression in a YTHDF2‐dependent manner, positioning the ALKBH5/YTHDF2–ELK1 axis as a promising target for OP diagnosis and therapy.

Nevertheless, certain limitations warrant consideration. Although we delineate the role of this regulatory axis in osteogenesis, ALKBH5 likely modulates additional m^6^A‐modified transcripts contributing to OP pathogenesis beyond ELK1. Future epitranscriptomic profiling (e.g., m^6^A‐seq) will be essential to uncover the broader network of ALKBH5‐dependent osteogenic regulation. In addition, the limited clinical sample size may restrict the generalizability of our results, necessitating validation in larger and more diverse cohorts. Moreover, our study did not explore the downstream targets of ELK1 in osteogenesis, which warrants further investigation to fully unravel the mechanistic cascade. Furthermore, the use of an adenoviral delivery system, which is known to have potential for off‐target effects and immune activation. Although we observed a clear phenotypic rescue, we did not directly measure immune responses and thus cannot fully rule out potential confounding effects. Future work utilizing bone‐specific delivery systems, such as adeno‐associated viruses (AAVs) or nanoparticles, will be important to confirm the specificity of our findings and minimize potential immunogenicity. We will keep investigating these limitations in our further research.

## Author Contributions

Huan Yu and Yongxing Peng conceived the study; Huan Yu and Ting Ruan conducted the experiments; Ting Ruan analyzed the data; and Huan Yu was a major contributor in writing the manuscript.

## Funding

The authors have nothing to report.

## Disclosure

All authors read and approved the final manuscript.

## Ethics Statement

The study was approved by the Ethics Committee of Jiangxi Provincial People’s Hospital, First Affiliated Hospital of Nanchang Medical College. Written informed consent was obtained from all patients. All experiments were performed in accordance with relevant guidelines and regulations.

## Conflicts of Interest

The authors declare no conflicts of interest.

## Supporting Information

Supporting Figure 1. ALKBH5 overexpression inhibited the osteogenic differentiation in hBMSCs. (A and B) mRNA and protein levels of ALKBH5 after the transfection of ALKBH5 overexpression vectors were analyzed by qPCR and western blot (*n* = 3). (C) Cell viability in each group was evaluated by CCK‐8 assay (*n* = 3). (D) ALP activity was detected by an ALP activity kit (*n* = 3). (E) Calcium nodules were observed using an ARS kit (*n* = 3). Student’s *t* test (A and B) or one‐way ANOVA (C–E) was used for statistical analysis. Data are expressed as mean ± SD. All *n* values represent the biological replicates.

## Supporting information


**Supporting Information** Additional supporting information can be found online in the Supporting Information section.

## Data Availability

The datasets generated and analyzed during the current study are available from the corresponding author upon reasonable request. This includes the primary data underlying the findings, such as raw qPCR values, micro‐CT image files and their quantitative analyses, uncropped western blot images, and source data for graphs. The data will be provided in a format that facilitates reuse.

## References

[1] Arceo-Mendoza R. and Camacho P. , Postmenopausal Osteoporosis: Latest Guidelines, Endocrinology and Metabolism Clinics of North America. (2021) 50, no. 2, 167–178, 10.1016/j.ecl.2021.03.009. 34023036

[2] Kerschan-Schindl K. , Prevention and Rehabilitation of Osteoporosis, Wiener Medizinische Wochenschrift. (2016) 166, no. 1-2, 22–27, 10.1007/s10354-015-0417-y, 2-s2.0-84958112061. 26769298

[3] Srivastava M. and Deal C. , Osteoporosis in Elderly: Prevention and Treatment, Clinics in Geriatric Medicine. (2002) 18, no. 3, 529–555, 10.1016/s0749-0690(02)00022-8, 2-s2.0-0036667602. 12424871

[4] Bandeira L. , Lewiecki E. , and Bilezikian J. , Romosozumab for the Treatment of Osteoporosis, Expert Opinion on Biological Therapy. (2017) 17, no. 2, 255–263, 10.1080/14712598.2017.1280455, 2-s2.0-85009944165. 28064540

[5] Yang J. G. , Sun B. , Wang Z. et al., Exosome-Targeted Delivery of METTL14 Regulates NFATc1 m6A Methylation Levels to Correct Osteoclast-Induced Bone Resorption, Cell Death and Disease. (2023) 14, no. 11, 10.1038/s41419-023-06263-4. PMC10643436 37957146

[6] Tian C. , Huang Y. , Li Q. , Feng Z. , and Xu Q. , Mettl3 Regulates Osteogenic Differentiation and Alternative Splicing of Vegfa in Bone Marrow Mesenchymal Stem Cells, International Journal of Molecular Sciences. (2019) 20, no. 3, 10.3390/ijms20030551, 2-s2.0-85060803634. PMC6387109 30696066

[7] Chen L. S. , Zhang M. , Chen P. et al., The m(6)A Demethylase FTO Promotes the Osteogenesis of Mesenchymal Stem Cells by Downregulating PPARG, Acta Pharmacologica Sinica. (2022) 43, no. 5, 1311–1323, 10.1038/s41401-021-00756-8. 34462564 PMC9061799

[8] Huang Y. , Wang S. , Hu D. , Zhang L. , and Shi S. , ALKBH5 Regulates Etoposide-Induced Cellular Senescence and Osteogenic Differentiation in Osteoporosis Through Mediating the m(6)A Modification of VDAC3, Scientific Reports-UK. (2024) 14, no. 1, 10.1038/s41598-024-75033-9. PMC11461877 39379688

[9] Li Z. , Wang P. , Li J. et al., The N(6)-Methyladenosine Demethylase ALKBH5 Negatively Regulates the Osteogenic Differentiation of Mesenchymal Stem Cells Through PRMT6, Cell Death and Disease. (2021) 12, no. 6, 10.1038/s41419-021-03869-4. PMC8178363 34088896

[10] Dp D. , Ets Oncogene Family, Indian Journal of Experimental Biology. (1997) 5, no. 4, 315–322. 9315229

[11] Wang Y. , Wang K. , Hu Z. et al., MicroRNA-139-3p Regulates Osteoblast Differentiation and Apoptosis by Targeting ELK1 and Interacting With Long Noncoding RNA ODSM, Cell and Death Disease. (2018) 9, no. 11, 10.1038/s41419-018-1153-1, 2-s2.0-85055802752. PMC6208413 30382082

[12] Liu Y. , Zhu J. , Wang W. H. et al., Exosomal Lncrna HCP5 Derived From Human Bone Marrow Mesenchymal Stem Cells Improves Chronic Periodontitis by miR-24-3p/HO1/P38/ELK1 Pathway, Heliyon. (2024) 10, no. 14, 10.1016/j.heliyon.2024.e34203. PMC11298838 39104492

[13] Li P. , Shi Y. , Gao D. et al., ELK1-Mediated YTHDF1 Drives Prostate Cancer Progression by Facilitating the Translation of Polo-Like Kinase 1 in an m6A Dependent Manner, International Journal of Biological Sciences. (2022) 18, no. 16, 6145–6162, 10.7150/ijbs.75063. 36439881 PMC9682537

[14] Hwang J. H. , Park Y. S. , Kim H. S. et al., Yam-Derived Exosome-Like Nanovesicles Stimulate Osteoblast Formation and Prevent Osteoporosis in Mice, Journal of Controlled Release. (2023) 355, 184–198, 10.1016/j.jconrel.2023.01.071. 36736431

[15] Chai S. , Yang Y. , Wei L. et al., Luteolin Rescues Postmenopausal Osteoporosis Elicited by OVX Through Alleviating Osteoblast Pyroptosis via Activating PI3K-AKT Signaling, Phytomedicine. (2024) 128, 10.1016/j.phymed.2024.155516. 38547625

[16] Zhang Y. , Bai J. , Xiao B. , and Li C. , BMSC-Derived Exosomes Promote Osteoporosis Alleviation via M2 Macrophage Polarization, Molecular Medicine. (2024) 30, no. 1, 10.1186/s10020-024-00904-w. PMC11577737 39563244

[17] He Q. , Liu Z. , Xia X. et al., Amlexanox Enforces Osteogenic Differentiation and Bone Homeostasis Through Inhibiting Ubiquitin-Dependent Degradation of Beta-Catenin, International Journal of Biological Sciences. (2024) 20, no. 13, 5254–5271, 10.7150/ijbs.101507. 39430247 PMC11489180

[18] Soave C. , Ducker C. , Kim S. et al., Identification of ELK1 Interacting Peptide Segments in the Androgen Receptor, Biochemical Journal. (2022) 479, no. 14, 1519–1531, 10.1042/bcj20220297. 35781489 PMC9422957

[19] Feng L. , Fan Y. , Zhou J. , Li S. , and Zhang X. , The RNA Demethylase ALKBH5 Promotes Osteoblast Differentiation by Modulating Runx2 mRNA Stability, FEBS Letters. (2021) 595, no. 15, 2007–2014, 10.1002/1873-3468.14145. 34105773

[20] Weng J. , Fan H. , Liu H. , Tang S. , and Zheng Y. , Abnormal Decrease of Macrophage ALKBH5 Expression Causes Abnormal Polarization and Inhibits Osteoblast Differentiation, Stem Cells International. (2023) 2023, 10.1155/2023/9974098. PMC10372297 37519314

[21] Li L. , Han M. , Li S. , Wang L. , and Xu Y. , Cyclic Tensile Stress During Physiological Occlusal Force Enhances Osteogenic Differentiation of Human Periodontal Ligament Cells via ERK1/2-Elk1 MAPK Pathway, DNA and Cell Biology. (2013) 32, no. 9, 488–497, 10.1089/dna.2013.2070, 2-s2.0-84882994341. 23781879 PMC3752521

[22] Shao Y. , Liu Z. , Song X. et al., ALKBH5/YTHDF2-Mediated m6A Modification of circAFF2 Enhances Radiosensitivity of Colorectal Cancer by Inhibiting Cullin Neddylation, Clinical and Translational Medicine. (2023) 13, no. 7, 10.1002/ctm2.1318. PMC10307995 37381158

[23] Xiao J. , Cai X. , Wang R. , Zhou W. , and Ye Z. , ALKBH5-YTHDF2 m6A Modification Axis Inhibits Rheumatoid Arthritis Progression by Suppressing NLRP3, Biochemical and Biophysical Research Communications. (2023) 668, 70–76, 10.1016/j.bbrc.2023.05.087. 37244037

