# Supplementary figures and images for: ALKBH5/YTHDF2 Axis Regulates Osteogenic Differentiation Through Mediating the m6A Modification of ELK1
Source: Int J Endocrinol. 2026 Jan 30;2026:2669506. doi: 10.1155/ije/2669506 (PMC12858426; doi:10.1155/ije/2669506)

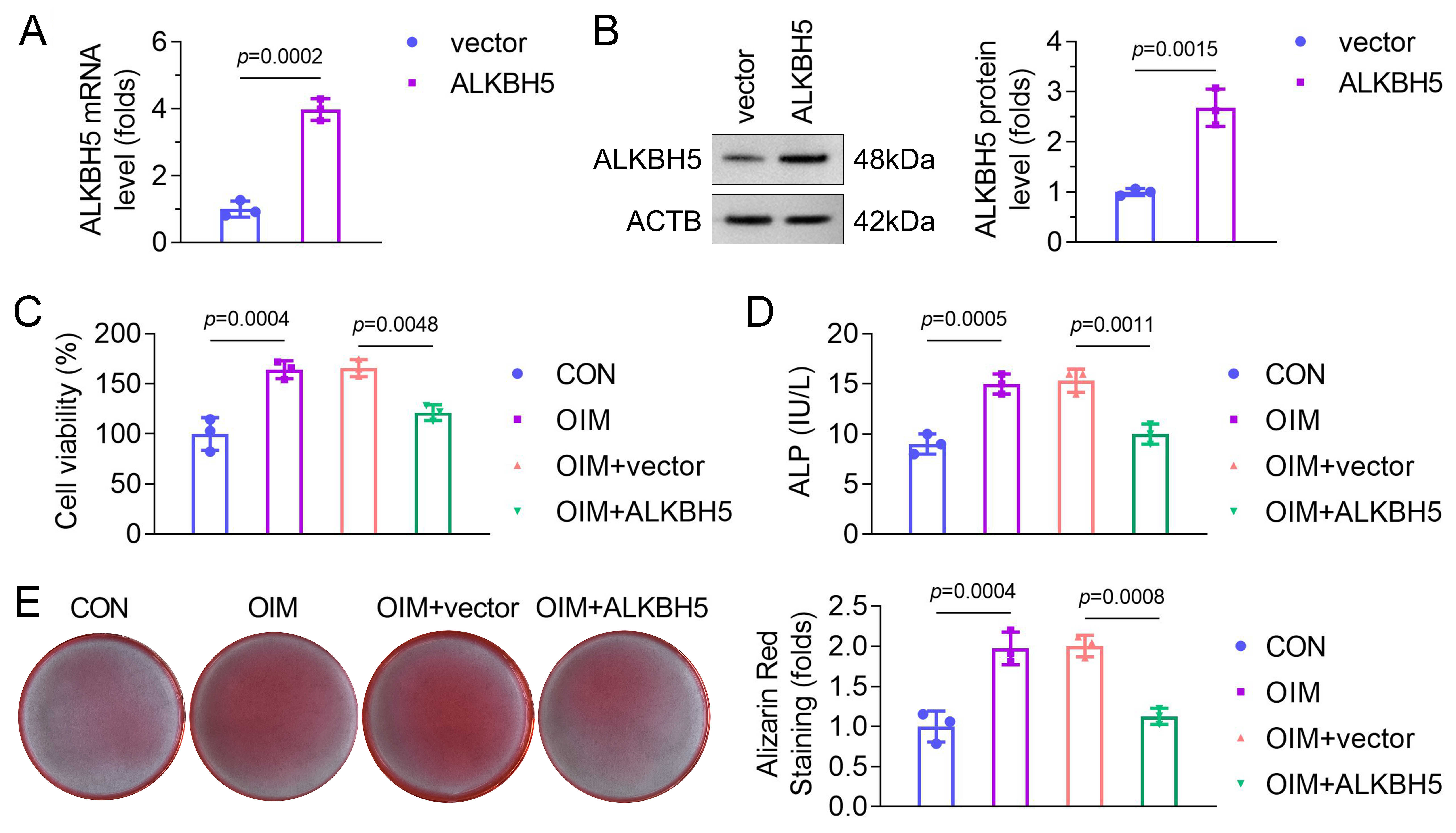

Supplement: Supplementary file 1 — Supporting Information Additional supporting information can be found online in the Supporting Information section. [file IJE-2026-2669506-s001.jpg]
